# Simultaneous testing of rule- and model-based approaches for runs of homozygosity detection opens up a window into genomic footprints of selection in pigs

**DOI:** 10.1186/s12864-022-08801-4

**Published:** 2022-08-06

**Authors:** Jan Berghöfer, Nadia Khaveh, Stefan Mundlos, Julia Metzger

**Affiliations:** 1 Research Group Veterinary Functional Genomics, Max Planck Institute for Molecular Genetics, Berlin, Germany; 2 Department of Biology, Chemistry and Pharmacy, Institute of Chemistry and Biochemistry, Freie Universität Berlin, Berlin, Germany; 3 Institute of Animal Breeding and Genetics, University of Veterinary Medicine Hannover, Hannover, Germany; 4 Research Group Development & Disease, Max Planck Institute for Molecular Genetics, Berlin, Germany; 5 Institute of Medical Genetics and Human Genetics, Charité-Universitätsmedizin Berlin, Berlin, Germany; 6 Charité-Universitätsmedizin Berlin, BCRT, Berlin Institute of Health Center for Regenerative Therapies, Berlin, Germany

**Keywords:** Runs of homozygosity, SNP density, Selection signatures, Pig genome

## Abstract

**Background:**

Past selection events left footprints in the genome of domestic animals, which can be traced back by stretches of homozygous genotypes, designated as runs of homozygosity (ROHs). The analysis of common ROH regions within groups or populations displaying potential signatures of selection requires high-quality SNP data as well as carefully adjusted ROH-defining parameters. In this study, we used a simultaneous testing of rule- and model-based approaches to perform strategic ROH calling in genomic data from different pig populations to detect genomic regions under selection for specific phenotypes.

**Results:**

Our ROH analysis using a rule-based approach offered by PLINK, as well as a model-based approach run by RZooRoH demonstrated a high efficiency of both methods. It underlined the importance of providing a high-quality SNP set as input as well as adjusting parameters based on dataset and population for ROH calling. Particularly, ROHs ≤ 20 kb were called in a high frequency by both tools, but to some extent covered different gene sets in subsequent analysis of ROH regions common for investigated pig groups. Phenotype associated ROH analysis resulted in regions under potential selection characterizing heritage pig breeds, known to harbour a long-established breeding history. In particular, the selection focus on fitness-related traits was underlined by various ROHs harbouring disease resistance or tolerance-associated genes. Moreover, we identified potential selection signatures associated with ear morphology, which confirmed known candidate genes as well as uncovered a missense mutation in the *ABCA6* gene potentially supporting ear cartilage formation.

**Conclusions:**

The results of this study highlight the strengths and unique features of rule- and model-based approaches as well as demonstrate their potential for ROH analysis in animal populations. We provide a workflow for ROH detection, evaluating the major steps from filtering for high-quality SNP sets to intersecting ROH regions. Formula-based estimations defining ROHs for rule-based method show its limits, particularly for efficient detection of smaller ROHs. Moreover, we emphasize the role of ROH detection for the identification of potential footprints of selection in pigs, displaying their breed-specific characteristics or favourable phenotypes.

**Supplementary Information:**

The online version contains supplementary material available at 10.1186/s12864-022-08801-4.

## Background

For centuries, traits under selection have been studied based on their phenotypic expression. Nowadays, the advent of molecular genetics and sequencing technologies allows examining the genomic background of any selected trait [1]. Studying runs of homozygosity (ROHs) has become the state-of-the-art method to detect signatures of selection and estimate inbreeding in domestic animal populations [2, 3]. ROHs are tracts of consecutive, homozygous genotypes, which are composed of two identical haplotypes inherited from a common ancestor [4, 5]. Longer ROHs likely result from recent inbreeding, whereas shorter ROHs indicate past inbreeding events, i.e., distant consanguinity or population founder effects [6, 7]. ROHs are non-randomly distributed across the genome and accumulate in highly inbred genomic regions known as ROH regions (ROHRs) [8, 9]. ROHRs represent shared regions of consecutive homozygous genotypes within or across populations and thus likely represent signatures of selection pressure leaving behind a local reduction in haplotype diversity, stretches of homozygous loci as well as reduced recombination rates [8–11]. The analysis of ROHRs particularly confers the advantage of identifying genomic regions under potential selection for favourable or unfavourable traits and involved genes [7, 8, 12].

Common approaches call ROHs based on single nucleotide polymorphisms (SNPs) detected by using SNP microarrays or whole genome sequencing (WGS) data [2, 13]. Microarrays have been used frequently for investigating homozygosity, although having limitations with regard to the detection of rare variants or the accurate identification of ROHs shorter than 1 Mb [4, 13]. With the continuously dropping costs for sequencing and availability of comprehensive genome assemblies, WGS has become the most advanced variant calling technology [2, 14]. As every accessible base can be called, WGS allows the detection of a comparatively high number of SNPs and particularly facilitates accurate detection of shorter ROHs [13]. Several factors constrain the quality of the ROH calling process, e.g., copy number variants (CNVs) or coverage gaps may introduce biases in the ROH analysis [15]. ROHs are more prevalent in genomic regions with a low recombination rate and high linkage disequilibrium (LD), particularly on the X chromosome or near centromeres [8, 16]. Subsequently, discrimination of homozygous segments into those caused by selection or induced by LD effects becomes increasingly difficult the smaller the segments are [17]. Furthermore, small inversions that prevent recombination or population demographic processes, e.g., bottlenecks or genetic drift, can also result in the formation of ROHs [4].

The detection of ROHs can be done using either a rule-based approach, screening for contiguous runs of homozygous genotypes in defined windows [18], or a model-based approach running a hidden Markov model (HMM) [19]. To avoid biases in further downstream analysis, ROH detection requires a closer look into the planned study-sample set and genotyping method to run the most suitable approach and apply the best fitting parameters [18, 20]. In particular, for the sliding-window approach applied by PLINK, the most commonly used rule-based tool to analyse inbreeding and genomic regions under selection in livestock [2, 21], a systematic customizing of ROH calling parameters is essential [13, 22]. Divers options and diverging configurations offered by PLINK for ROH prediction were supposed to make it difficult to compare the results of different studies [23, 24]. To address this issue, different suggestions have been made on how to apply ROH analysis parameters in domestic animal populations using the rule-based approach offered by PLINK [6, 7, 22, 25]. In contrast, model-based approaches rely on likelihood-ratio tests accounting for marker allele frequencies and genotyping errors in predefined window sizes [20]. Genomes are modelled as a mosaic of HBD and non-HBD segments and assigned to a single class as applied by BCFtools/RoH [26] or, to better fit individual genetic data and thus for a more accurate estimation, allocated to multiple HBD classes as applied by RZooRoH [19, 20, 27]. These tools have been particularly used for estimations of inbreeding levels in livestock so far [27].

Recent studies in pigs identified selection signatures and candidate genes related to economically important traits such as growth, reproduction, and meat quality [14, 28–31] or adaptability, disease resistance and immunity [14, 29, 30] in Chinese and Western breeds. ROHRs have been used to analyse selection signatures and genes linked to the phenotypic characteristics of Diannan small-ear pigs [32], Laiwu pigs [17], Mangalitza pigs [33], micro pigs [34], Piétrain pigs [35], Sicilian pigs [36], Xidu black pig [37] and different European autochthonous and commercial pigs [8, 9, 34, 35, 38–40]. Previous studies in pigs were focussed mainly on longer ROHs and optimized the ROH calling parameters accordingly. However, it was proposed that without further adjustment of the parameters, ROH calling algorithms might miss shorter ROHs in less inbred animals and thus confound the further downstream analysis [6, 13].

In this study, we provide a simultaneous testing of rule- and model-based approaches as well as a downstream systematic parameter testing for the accurate analysis and detection of ROHs in pigs using high confidence SNPs predicted from WGS data. We highlight the impact of ROH detection methods, individual parameters, their dependencies from each other, and the consequences of shifting individual or multiple factors. Hereby, we suggest a workflow for the detection of ROHRs and emphasize the limitations of ROH detection methods, particularly with regard to the detection of different size classes of ROHs. The aim of this study is to provide a strategic ROHRs detection in order to identify signatures of potential selection for breed-characteristics and favourable genotype–phenotype effects in pigs.

## Results

### Whole genome sequencing

**Fig. 1:**
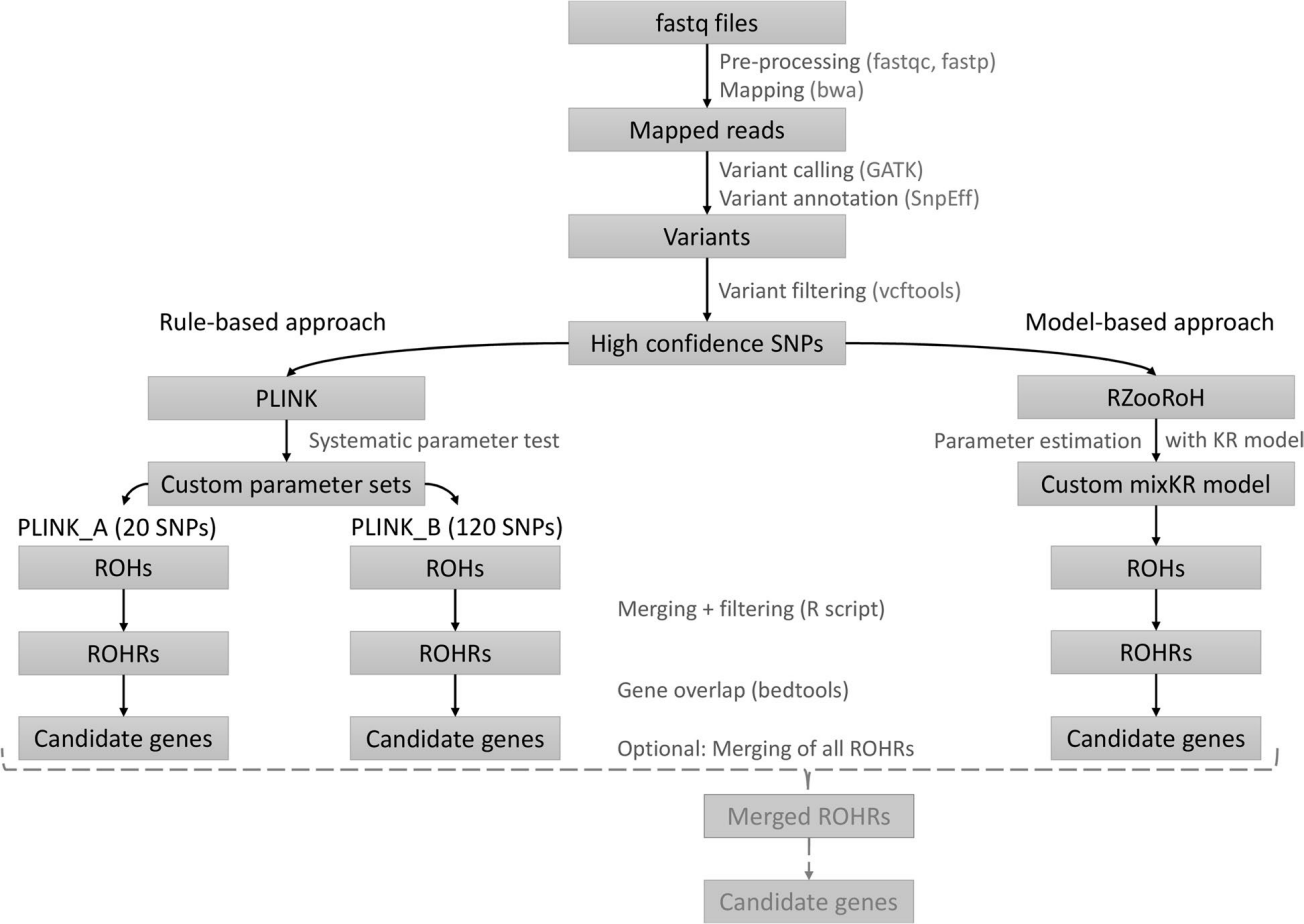
Workflow of ROH detection pipeline. Fastq files were used as input, variants were called, quality controlled and underwent ROH detection with the rule-based ROH detection approach implemented in PLINK and the model-based ROH detection approach run by RZooRoH. Finally, ROHRs were identified and investigated for potential genes of interest using functional enrichment analysis. A ROHRs-merging step was run as optional for particular applications

A step-by-step workflow was developed for processing WGS-fastq files up to final ROHR analysis (Fig. 1).

First of all, mapping of whole-genome sequencing data from 14 pigs from different populations, and six different crossbreds was performed with stringent quality parameters. This resulted in a coverage of 15.67X-46.32X, an error rate of 0.64% to 1.49% (mismatches/bases mapped) and an average mapping quality score of 34.9–36.7. Variant calling along with all 20 different pigs revealed 37,566,351 SNPs and 8,564,529 insertions or deletions (INDELs).

### Identification of high-quality SNPs

**Fig. 2:**
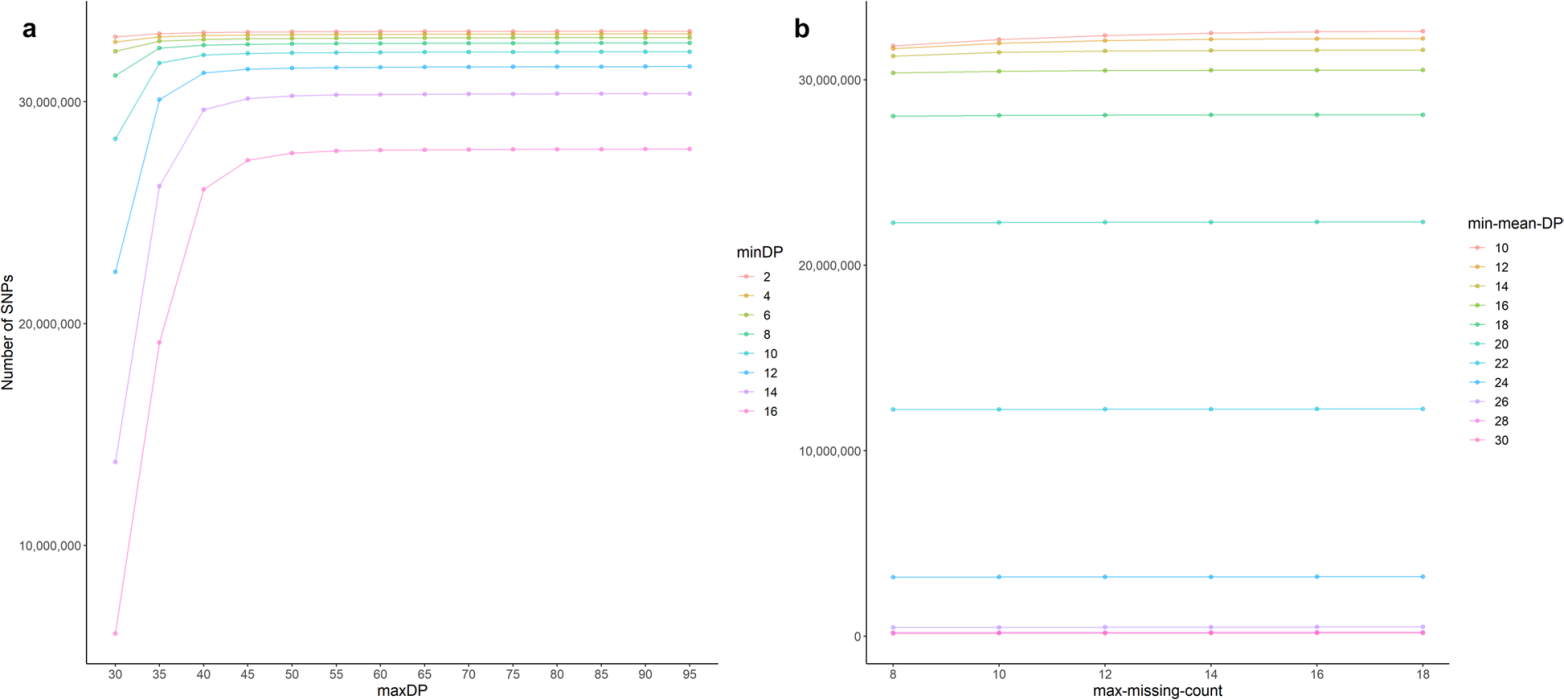
Evaluation of SNP filtering parameters.** a** The number of SNPs after filtering with the minimum read depth (minDP, rainbow colors), the maximum read depth (maxDP) and a minimum quality threshold (minQ) of 30 are displayed. An increase of the number of filtered SNPs can be observed with an increasing maxDP and a decrease with a higher minDP. **b** The number of SNPs resulting from strategic testing of minimum mean read depth (min-meanDP) and maximum number of allowed missing genotypes (max-missing-count) testing. An increase of the number of filtered SNPs is displayed relative to an increasing number of allowed missing genotypes (max-missing-count) and a lower minimum mean read depth (min-meanDP)

For the identification of a high-quality SNP set, variant quality parameters were tested for their efficiency based on the underlying dataset. Thus, the number of called SNPs for a minimum and maximum read depth (*minDP/maxDP*) with a fixed minimum quality threshold (*minQ*) of 30 were evaluated in a first step. Higher values of *minDP* resulted in a significantly lower number of SNPs (Fig. 2a);

For example, *minDP* 2 resulted 32,933,744 SNPs, whereas *minDP* 16 provided 6,020,022 SNPs (Additional file 1: Table S1). Accordingly, we observed the highest number of SNPs (33,184,918) when *minDP* 2 and *maxDP* 95 were applied. Upscaling *minDP* up to 6 only slightly decreased the number of SNPs and resulted in the same size range as *minDP* 2. In general, we found that scaling up *maxDP* increased the total number of SNPs. This increase was particularly important up to *maxDP* 40 (especially for *minDP* > 10) and moderate up to *maxDP* 95.

In a next step, the parameter *min-meanDP* was tested using a large SNP set obtained from *minDP* 6, *maxDP* 95, *minQ* 30 implementation. We observed a decrease in the total number of SNPs independent of the additionally applied parameter *max-missing-count* (Fig. 2b). A *min-meanDP* of 10–14 resulted in an only slightly reduced number of SNPs, but values above 16 showed a significant reduction of filtered SNPs. We obtained the highest number of SNPs for a *min-meanDP* 10 (32,613,313 SNPs based on *max-missing-count* 18) and the lowest number of SNPs for a *min-meanDP* 30. However, compared to the highest number of SNPs filtered with *min-meanDP* 10, we obtained an even higher number of SNPs filtering only based on *minDP* and *maxDP* using the same parameter settings (32,900,536 SNPs based on *max-missing-count* 18). Thus, the *min-meanDP* showed its dependence on the average read depth per SNP, which was > 14X in the majority of the investigated samples. Accordingly, a significant proportion of SNPs considered as high-quality SNPs in this dataset was removed by filtering when *min-meanDP* > 10 was applied. Next, *max-missing-count* was set as an additional parameter: The number of SNPs increased by 0.2% to 17.9% when *max-missing-count* 10 to 18 was applied relative to the results with *max-missing-count* 8, dependent on the tested combinations of *min-meanDP* (Additional file 2: Table S2). For *min-meanDP* 10, a *max-missing-count* from eight to 18 increased the number of filtered SNPs by 2.4% from 31,820,357 to 32,613,313 SNPs. However, the increase in the number of SNPs reached saturation at max-missing-count 15, which implied 75% of missing genotypes at maximum allowed in all 20 individuals. Based on these results, we identified *minDP* 6, *maxDP* 95, without *min-meanDP* restrictions, and max-missing-count 15 as optimal filtering conditions for our dataset.

### Configuration tests for rule-based approach

**Fig. 3:**
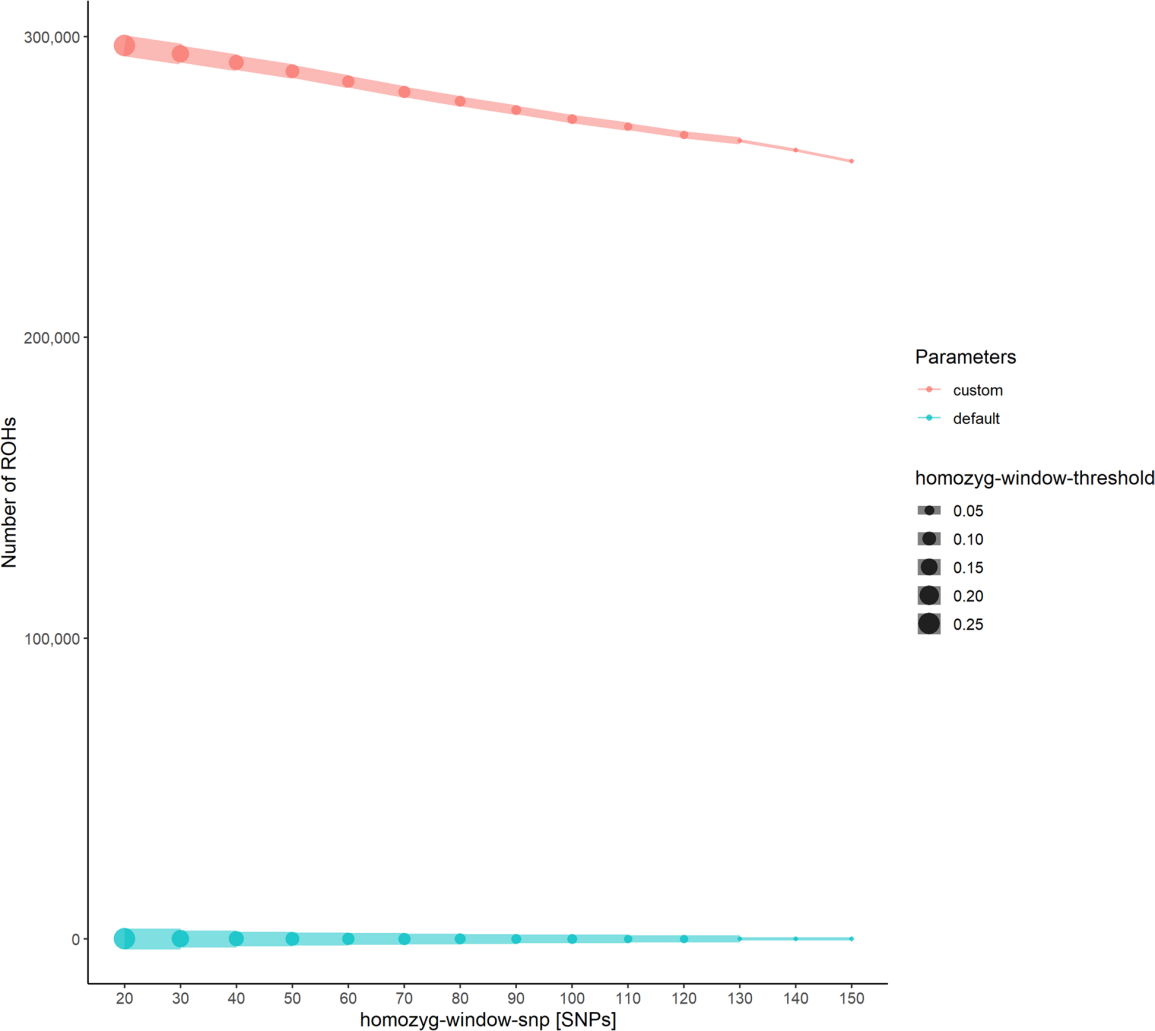
Configuration test for PLINK’s ROH detection parameters defining the scanning window. The number of detected ROHs for different scanning window sizes (homozyg-window-snp) and scanning window-thresholds (homozyg-window-threshold) are displayed. A higher number of ROHs was detected when a lower homozyg-window-snp value was applied. In general, the number of ROHs was markedly higher in custom parameter settings (red) compared to default parameter settings (blue)

Based on the filtered set of high-quality SNPs, we defined custom parameter settings for ROH detection using PLINK according to suggested formula and iterated individual parameters. In total, this custom set resulted in the detection of a higher number of ROHs for different scanning window sizes compared to PLINKs’ default settings. For both custom and default parameters, the number of detected ROHs decreased with an increasing scanning window size (*homozyg-window-snp*, Fig. 3).

From *homozyg-window-snp* 20 to 150, the number of ROHs dropped by 13% (relative to the number of ROH *homozyg-window-snp* 20) using the custom parameters and by 32% using default parameters (Additional file 3: Table S3). In addition, the average length of the detected ROHs decreased with a higher number of SNPs per scanning window. When custom parameters were applied, the average ROH length decreased from 37 kb (*homozyg-window-snp* 20) to 33 kb (*homozyg-window-snp* 150). With default settings, we found in total fewer ROHs that had a significantly higher average ROH length. The average ROH length decreased significantly with an increasing window size from 744 kb for *homozyg-window-snp* 20 to 498 kb for *homozyg-window-snp* 150. In general, the dependent parameter *homozyg-window-threshold* decreased with an increasing scanning window size as defined in the custom settings. However, a lower threshold resulted in a decreased average ROH length even with a larger scanning window size, as observed for a *homozyg-window-snp* 120 and a threshold of 0.03.

**Fig. 4:**
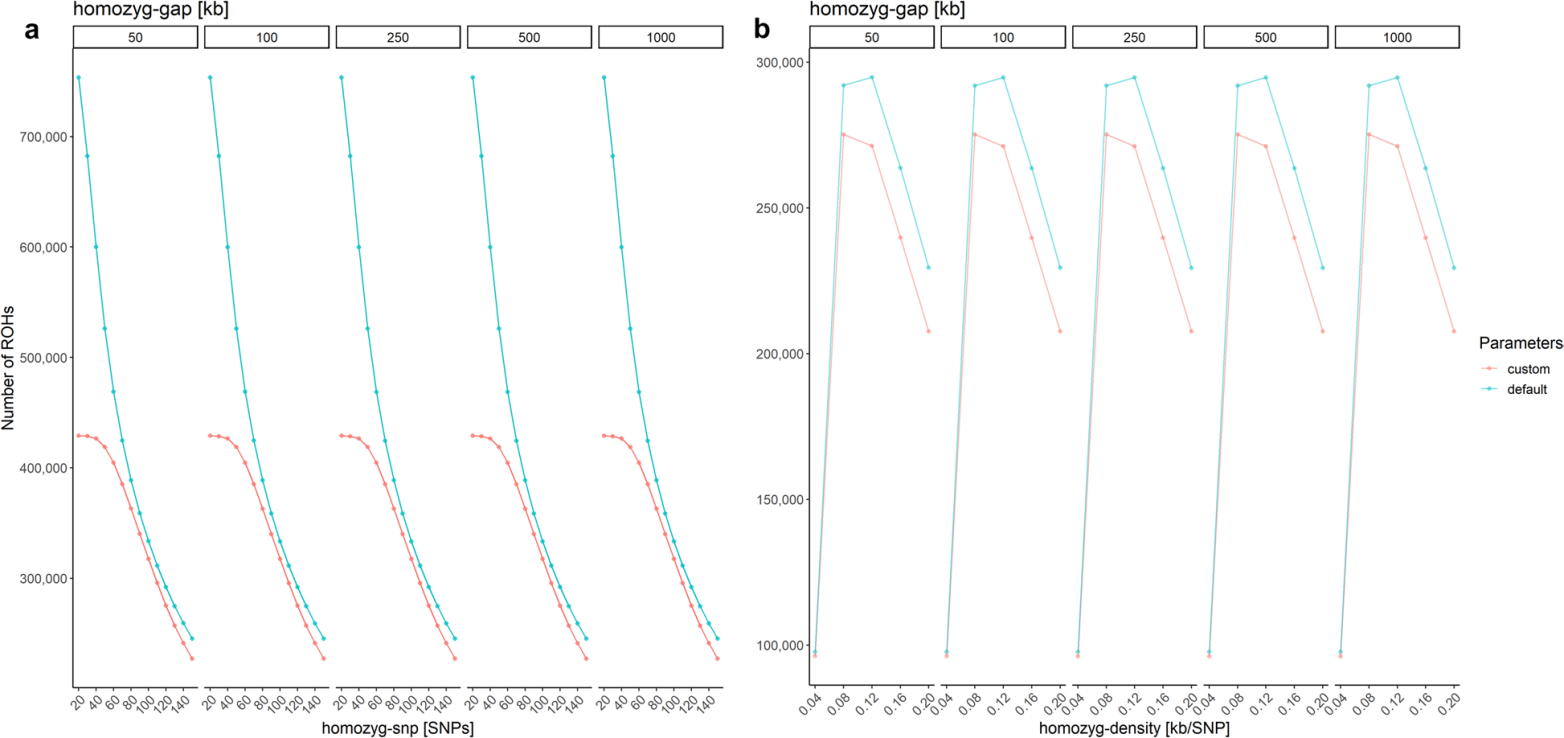
Test of PLINK’s ROH detection parameters defining a ROH segment.** a** The number of detected ROHs for different minimum SNP counts (homozyg-snp) and maximum gap sizes between two SNPs (homozyg-gap, on top of each plot) is displayed. A higher number of ROHs with a lower ROH length is detected by using the less stringent default parameter settings (blue). **b** Test settings for ROH detection based on SNP density. The number of detected ROHs for a maximum inverse density (homozyg-density) and the maximum gap size between two ROHs (homozyg-gap, on top of each plot) are given. The highest number of ROHs was identified for homozyg-density of 0.08 kb/SNP based on custom parameter settings (red) and homozyg-density of 0,12 kb/SNP for default parameter settings (blue)

Next, *homozyg-snp* was considered: The total number of ROHs decreased with an increasing minimum SNP count from 429,101 ROHs (*homozyg-snp* 20) to 227,138 ROHs (*homozyg-snp* 150) for *homozyg-gap* 50 using our custom parameters (Fig. 4a).

The same trend was observed for default parameter settings. However, with default parameters we found a significantly higher number of ROHs for *homozyg-snp* 20–80 compared to the more stringent custom parameters. For example, we detected 753,540 ROHs for *homozyg-snp* 20 (*homozyg-gap* 50) using default settings. However, with increasing ROH length the number of ROHs converged with the ones found based on custom parameters. Furthermore, for both custom and default parameters the average ROH length increased with a larger *homozyg-snp*. When custom parameters were applied, the average ROH length increased from 24.37 kb for *homozyg-snp* 20 to 37.44 kb for *homozyg-snp* 150 (*homozyg-gap* 50, Additional file 4: Table S4). Accordingly, with the default parameters, the average ROH length increased from 18.21 kb for *homozyg-snp* 20 to 40.67 kb for *homozyg-snp* 150 (*homozyg-gap* 50). In addition to the minimum SNP count, we evaluated the role of *homozyg-gap*. With an increasing *homozyg-gap* from 50 to 1,000, we observed minimal deviations in length and number of detected ROHs. From *homozyg-gap* 250 to 1000, the number of ROHs remained constant, however, only for homozyg-gap 50 to 250 the number of ROHs changed by less than 0.01%.

**Fig. 5:**
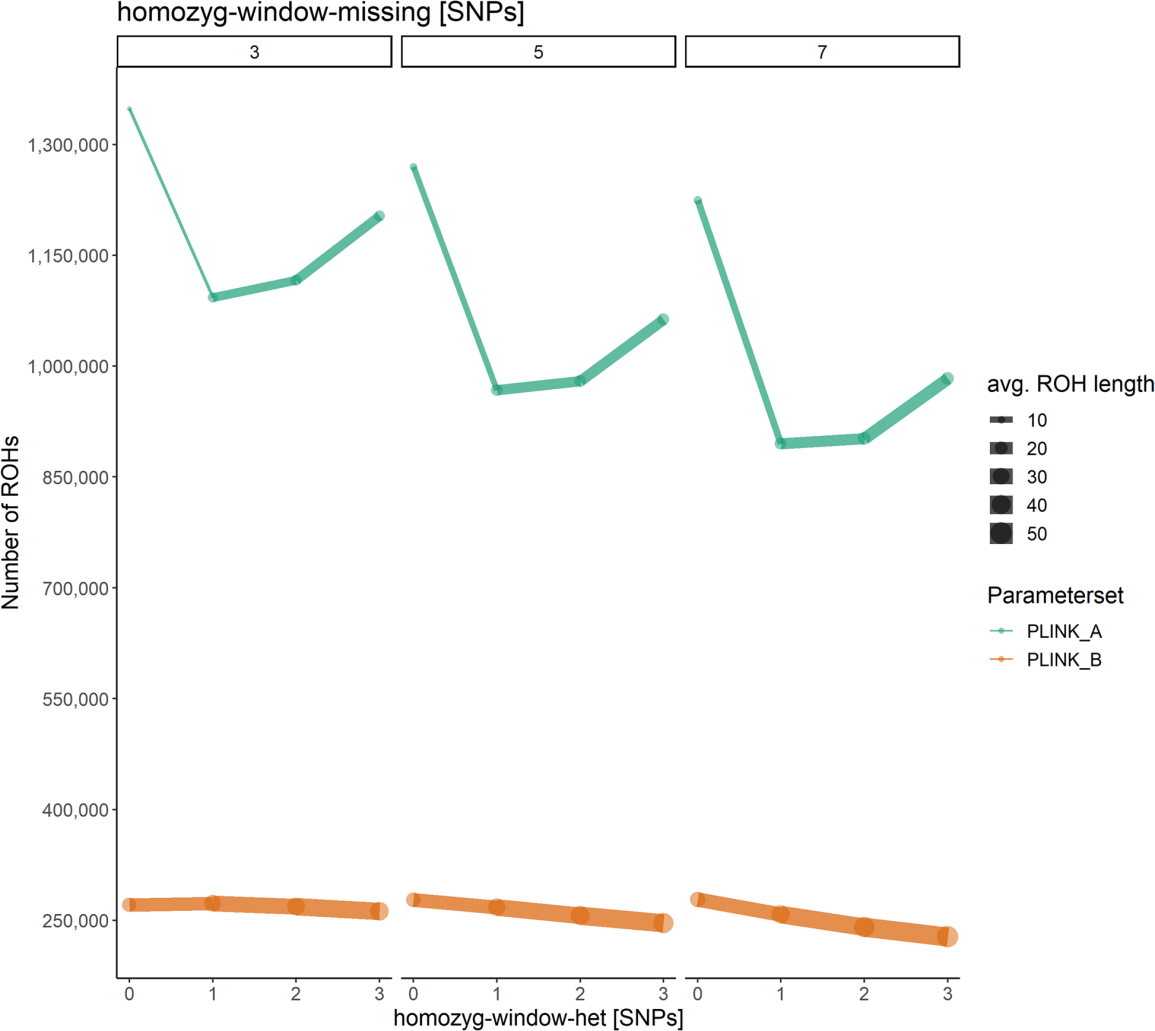
Evaluation of the consequences resulting from the number of heterozygous and missing SNPs in PLINK. The number of ROHs and average ROH length (in kb) dependent of the maximum number of heterozygous SNPs (homozyg-window-het) and missing SNPs allowed per window (homozyg-window-missing, on top of each plot) for parameter sets PLINK_A (homozyg-SNP 20, green) and PLINK_B (homozyg-SNP 120, orange) is displayed

In contrast, *homozyg-density* made a significant impact on the number of ROHs. Based on custom parameters, the number of ROHs increased significantly from the minimum density of 0.04 kb/SNP, reached a maximum of detected ROHs at 0.08 kb/SNP and dropped down when higher *homozyg-density* values were applied (Fig. 4b). We observed a similar trend for default settings that implemented a much smaller scanning window size (default: 50 SNPs vs. custom: 120 SNPs). Here, the number of ROHs increased from a minimum at 0.04 kb/SNP up to a maximum at 0.12 kb/SNP even higher than the maximum number of ROHs detected with custom parameters. With *homozyg-density* > 0.12 kb/SNP, the number of ROHs dropped significantly but still remained higher than the number of ROHs detected with custom parameters. Notably, in this test setting we detected much more ROHs using the default parameters (*homozyg-window-snp* 50, *homozyg-window-threshold* 0.05: 294,801 ROHs) compared to the results with custom parameters (*homozyg-window-snp* 120, *homozyg-window-threshold* 0.04: 275,230 ROHs). In contrast, the average ROH length increased with higher *homozyg-density* values for both custom and default parameter settings from < 20 kb/SNP with *homozyg-density* 0.04 to an average length of > 60 kb/SNP with *homozyg-density* 0.2 (homozyg-gap 50, Additional file 5: table S5). Based on these results, we identified two custom PLINK parameter sets subsequently called “PLINK_A” (*homozyg-snp* 20; specifically designed to include shorter ROHs) and “PLINK_B” (*homozyg-snp* 120; based on calculated values for this investigated dataset) optimized for the detection of ROHs in our dataset and ran further tests on *homozyg-window-het* and *homozyg-window-missing*. The maximum number of ROHs and the smallest average ROH length were detected provided that no heterozygous SNPs were allowed within a window (*homozyg-window-het* 0, Fig. 5).

Compared to this maximum, the total number of ROHs decreased when allowing one heterozygous SNP per window, whereas the average ROH length increased (*homozyg-window-het* 1). For PLINK_B, the number of ROHs detected for each parameter setting was closer to each another compared to PLINK_A (Additional file 5: Table S5). Nevertheless, for each tested value of PLINK_B, the average ROH length differed much stronger than for PLINK_A. However, with an increasing *homozyg-window-missing* and *homozyg-window-het*, the differences in the total number of ROHs became more pronounced. For both PLINK_A and PLINK_B, we observed the maximum of detected ROHs when allowing three missing calls per window (*homozyg-window-missing* 3). As the number of tolerated missing calls increased, the total number of ROHs decreased slightly, but the average ROH length remained unchanged. Subsequently, based on the obtained results, we finally defined two now optimized PLINK_A and PLINK_B for efficient ROH detection in our investigated dataset of 20 pigs (Table 1).

**Table 1:** Overview of parameter settings for SNP filtering and ROH detection with PLINK Custom PLINK parameter settings PLINK_A and PLINK_B adjusted to our investigated dataset for efficient subsequent ROH calling are displayed

Tool	Parameters	PLINK_A	PLINK_B
Vcftools	minDP	6	6
	maxDP	95	95
	max-missing-count	15	15
PLINK	homozyg-snp	20	120
	homozyg-kb	1.6	9.93
	homozyg-density	0.08	0.08
	homozyg-gap	1000	1000
	homozyg-window-snp	20	120
	homozyg-window-threshold	0.25	0.04
	homozyg-window-missing	5	5
	homozyg-window-het	1	1

### ROH detection and distribution among individual pigs

In the 20 different pig breeds/populations, we found for each pig different numbers of ROHs ranging from 29,743 ROHs in the Iberian pig to 108,549 ROHs in the Yorkshire pig (PLINK_A), from 525 ROHs in the Yorkshire pig to 22,079 ROHs in the Wuzhishan Minipig (PLINK_B) and from 17,800 in the Duroc pig up to 55,669 ROHs in the Meishan pig (RZooRoH).

To learn more about the characteristics of detected ROH within the individual pigs, the length distribution of ROHs was examined by assigning all ROHs to five length categories “0–20 kb”, “20–50 kb”, “50–250 kb”,“250–500 kb” and “ > 500 kb” (Additional file 6: Table S6) Assuming an average generation time of 2 years for all pigs [41, 42], the estimated ages of these ROH length categories roughly correspond to > 5000, 5000–2000, 2000–400, 400–200, 200–100, and > 100 years, respectively. In total, PLINK_A provided the highest sum of all ROH of all samples of 1,001,426 compared to 266,884 ROHs (PLINK_B) and 563,312 ROHs (RZooRoH). These ROHs from PLINK_A (average size 14.78 kb) were much shorter than those detected using RZooRoH (44.37 kb) and PLINK_B (34.45 kb). This was particularly visible considering the distribution among the determined length categories. For both PLINK parameter sets and RZooRoH, the highest number of ROHs was detected within the first three length categories “0–20 kb”, “20–50 kb” and “250–500 kb”.

### Comparison of ROH calling approaches

**Fig. 6:**
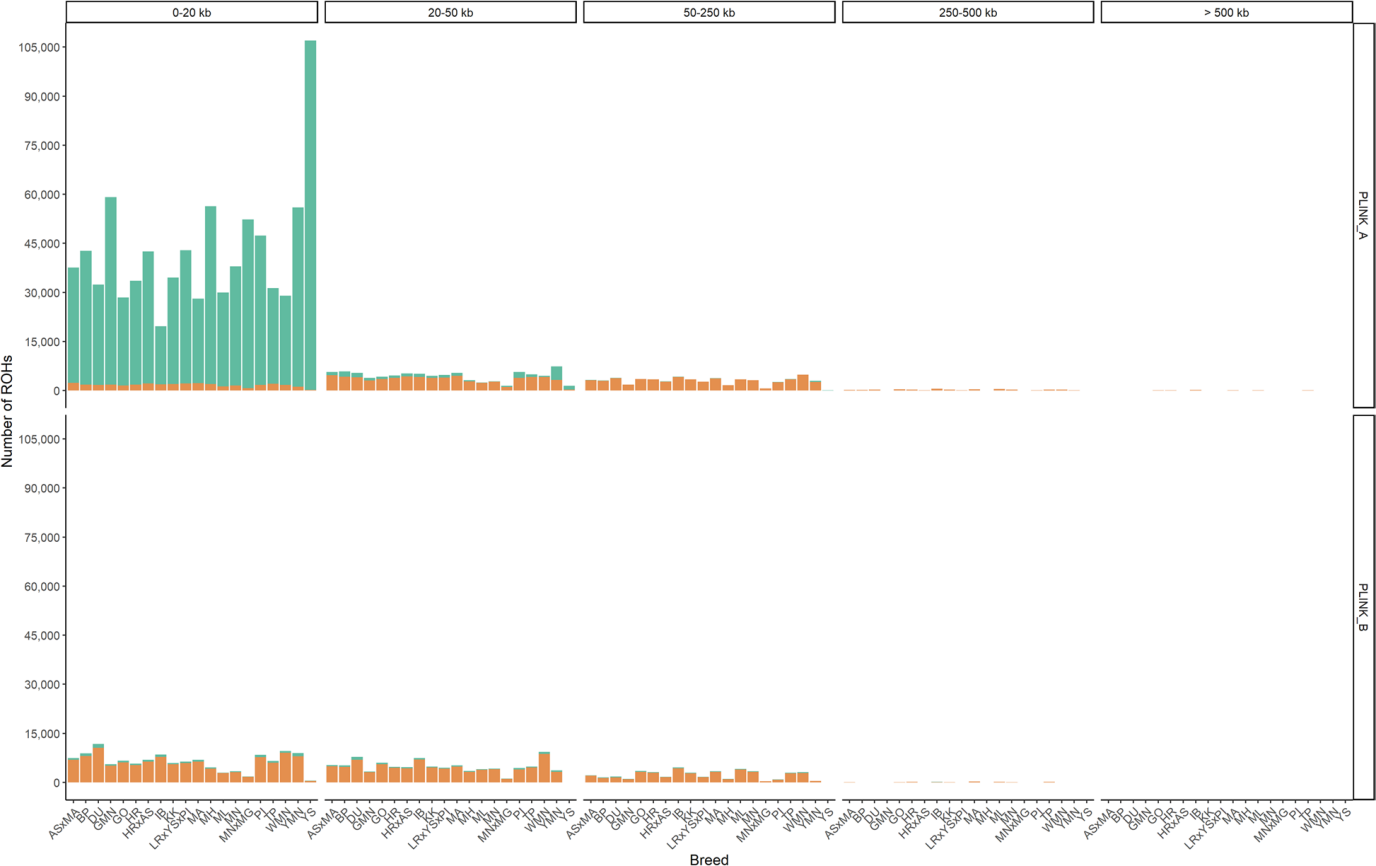
Size distribution and overlap of ROHs detected using PLINK_A and PLINK_B. For each parameter set (PLINK_A, PLINK_B), the number of ROHs per size category are displayed for each investigated breed (x-axis: ASxMA: Angeln Saddleback × Mangalitza, BP: Bentheim Black Pied, DU: Duroc, GMN: Goettingen Minipig, GO: Gloucester Old Spot, HR: Husum Red Pied, HRxAS: Husum Red Pied × Angeln Saddleback, IB: Iberian, KK: Kune, LR × YS × PI: Landrace × Yorkshire × Pietrain, MA: Mangalitza, MH: Meishan, ML: Mini-Lewe, MN: Minipig, MN × MG: Minipig × Mangalitza, PI: Pietrain, TP: Turopolje, WMN: Wuzhishan minipig, YMN: Yucatan miniature pig, YS: Yorkshire). All ROHs were assigned to size categories of “0–20 kb”, 20–50 kb”,”50–250 kb”,”250–500 kb”,”500–1000 kb”,” > 1000 kb” (indicated on top) and the proportion of ROHs overlapping with the other tool or parameter set was highlighted in orange and designated as “yes”

First of all, the comparison of PLINK parameter sets A and B revealed the majority of ROHs with 0–20 kb length called by PLINK_A to be unique to this dataset and thus not overlapping with ROHs detected using PLINK_B (Fig. 6).

This difference was particularly prominent for the Yorkshire pig, Yucatan miniature pig, and Goettingen Minipig. Considering PLINK_B, only a small proportion of ROHs with a size of 0–250 kb was unique to the detected ROHs of this parameter set, whereas the majority of ROHs overlapped with those detected using PLINK_A. In the other size classes “250–500 kb”, and “ > 500 kb”, far less ROHs were found and but more ROHs were detected using PLINK_A. Based on PLINK_A, almost all ROHs > 250 kb overlapped with ROH detected using PLINK_B. Moreover, for PLINK_B, the proportion of non-overlapping ROHs was far higher for ROHs 250–500 kb. Percentage of overlap per animal ranged from 1.31% for Yorkshire pigs to 44.06% for Kune Kune in PLINK_A and from 87.59% for Yucatan Minipig to 96.43% in Mini-Lewe in PLINK_B (Additional file 7: Table S7). In total, 87.59–96.43% of the ROHs detected using PLINK_B could also be detected using PLINK_A. Interestingly, we found the lowest number of ROHs per individual in PLINK_B for the Yorkshire pig with 525 ROHs, all with a size of 0–50 kb and of which 92% overlap with ROHs in PLINK_A. In contrast, in PLINK_A, we found the highest number of ROHs and thus the highest homozygosity per individual for the Yorkshire pig with 108,549 ROHs in the size range 0–250 kb, of which only 0.4% overlap with ROHs detected with PLINK_B.

**Fig. 7:**
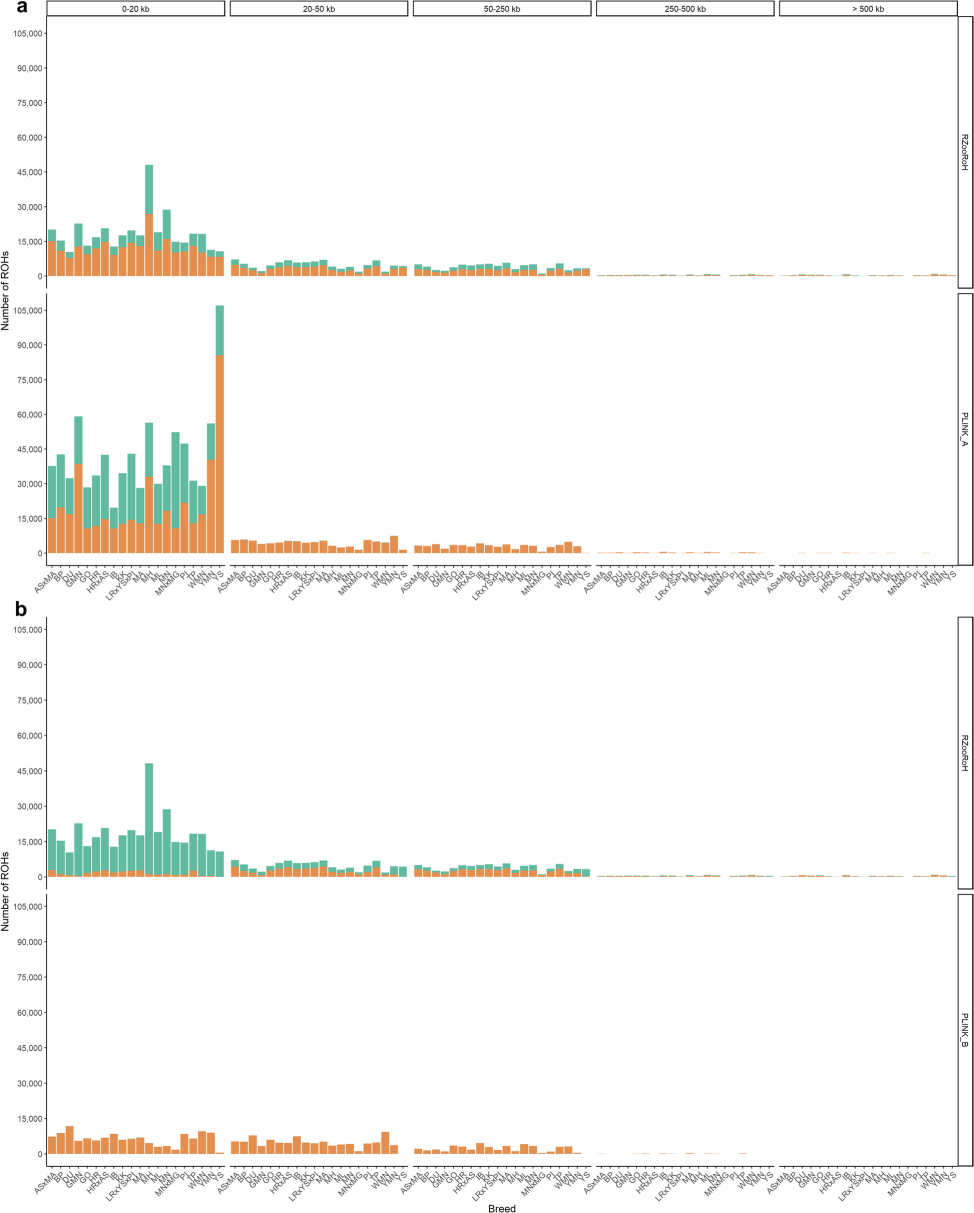
Size distribution and overlap of ROHs detected using RZooRoH and PLINK. For each tool and parameter set (PLINK_A, PLINK_B), the number of ROHs per size category are displayed for each investigated breed (x-axis: AS × MA: Angeln Saddleback × Mangalitza, BP: Bentheim Black Pied, DU: Duroc, GMN: Goettingen Minipig, GO: Gloucester Old Spot, HR: Husum Red Pied, HR × AS: Husum Red Pied × Angeln Saddleback, IB: Iberian, KK: Kune, LR × YS × PI: Landrace × Yorkshire × Pietrain, MA: Mangalitza, MH: Meishan, ML: Mini-Lewe, MN: Minipig, MN × MG: Minipig × Mangalitza, PI: Pietrain, TP: Turopolje, WMN: Wuzhishan minipig, YMN: Yucatan miniature pig, YS: Yorkshire). All ROHs were assigned to size categories of “0–20 kb”, “20–50 kb”, “50–250 kb”, “250–500 kb”, “500–1000 kb”, “ > 1000 kb” (indicated on top) and the proportion of ROHs overlapping with the other tool or parameter set was highlighted in orange and designated as “yes”

Furthermore, ROHs detected with PLINK parameter sets a and b were compared each with the results from RZooRoH ROH calling (Fig. 7).

**Fig. 8:**
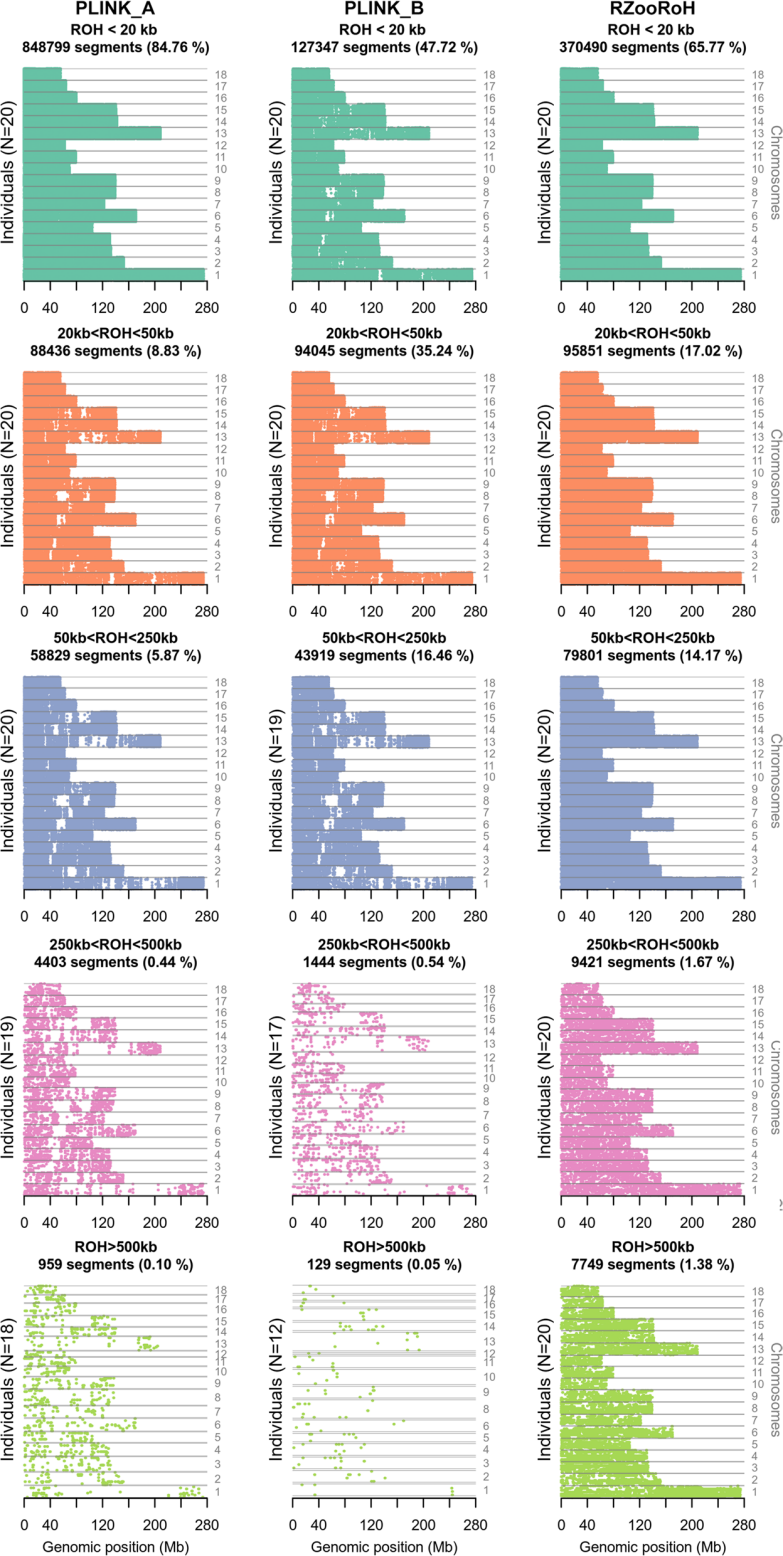
Chromosomal and size-distribution of ROHs detected using PLINK and RZooRoH. Each row contains ROH segments obtained from a single individual (if applicable) put on top of the ROH segments of another individual. These rows are grouped for all 20 individuals based on their position chromosomes 1 (bottom of each panel) to 18 (top of each panel). ROHs are displayed in 5 length categories: 0–20 kb, 20–50 kb, 50–250 kb, 250–500 kb and above 500 kb

A major overlap of ROHs obtained from RZooRoH was found in the size category of 0–20 kb with the ROHs from PLINK_A, whereas only a small proportion overlapped in this category with ROHs detected using PLINK_B. Within the range of > 20 kb unique ROHs neither called by using PLINK_A or PLINK_B in the output file from RZooRoH were particularly frequent. In total, the mean overlap of ROHs detected by RZooRoH with ROHs detected by PLINK was quite frequent (65.6%, PLINK_A), or low (24.7%, PLINK_B). Comparing RZooRoH ROH calling data with those identified using PLINK_A and PLINK_B across all size categories, the proportion of overlaps per animal ranged from 0.87% (Yorkshire) to 49.00% (Minipig × Mangalitza, PLINK_A) and 0.51% (Yorkshire) to 27.49% (Landrace × Yorkshire × Pietrain, PLINK_B). The other way around, comparing ROH calling results from PLINK’s analysis with those detected using RZooRoH revealed a mean overlap across all animals of 58.1% (PLINK_A) and 99.9% (PLINK_B). With regard to the individual pigs, PLINK ROH calling data revealed an intersection with ROHs detected by RZooRoH from 50.13% (Minipig × Mangalitza) to 95.17% (Wuzhishan minipig, PLINK_A) as well as from 98.62% (Minipig × Mangalitza) to 99.95% (Wuzhishan minipig, PLINK_B). Thus, RZooRoH identified the majority of ROHs detected by PLINK and above that, called further unique ROHs not identified by the rule-based approaches. The higher number of additional ROHs was particularly frequent in the larger ROH-size categories, which resulted in a more balanced distribution of called ROHs with regard to ROH length across all chromosomes and individuals (Fig. 8).

**Fig. 9:**
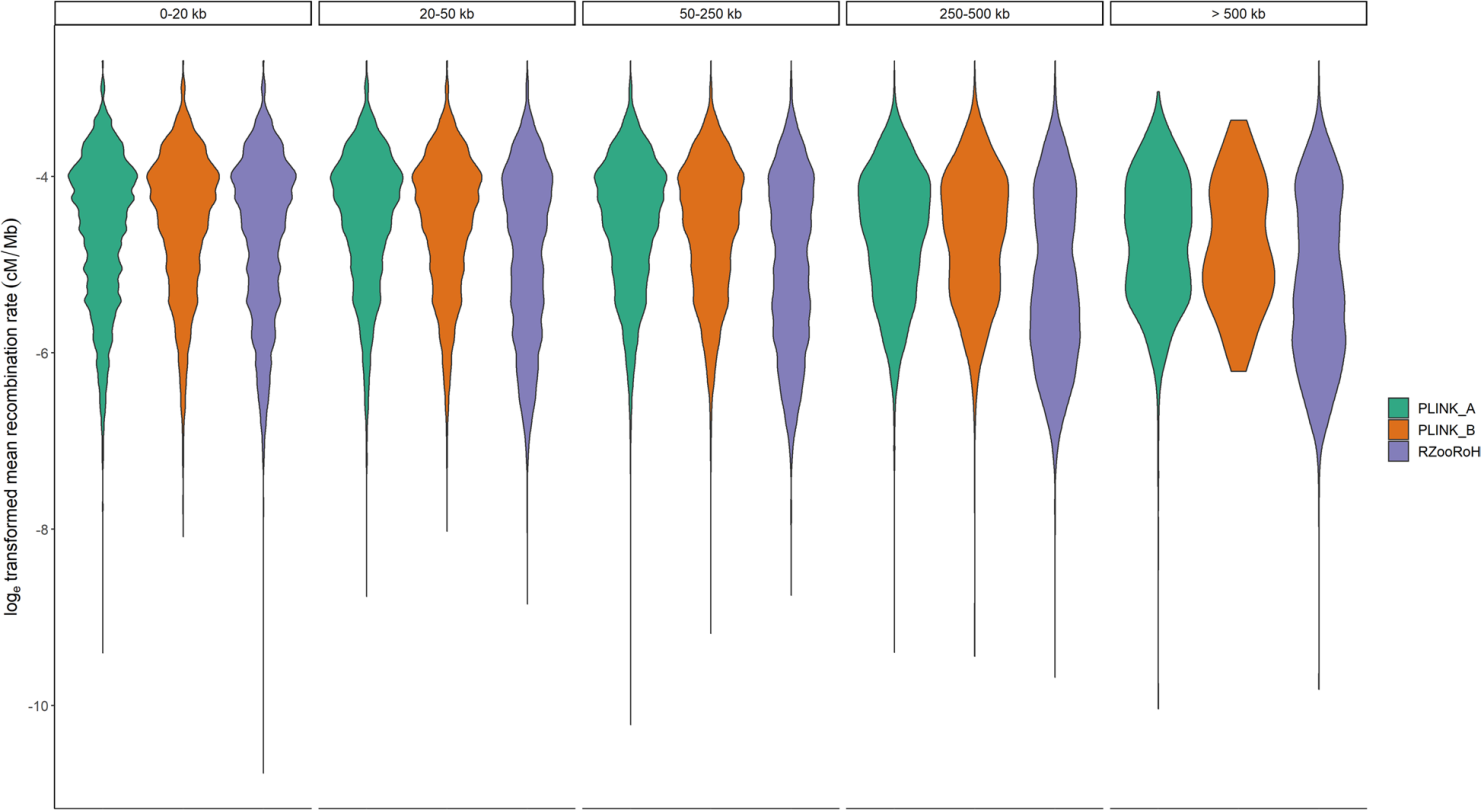
Relationship between distribution of ROHs lengths and recombination rates for PLINK and RZooRoH. For each tool and parameter set (indicated by colour: PLINK_A (orange) and PLINK_B (green), RZooRoH (purple)), the ROH density is displayed as a function of the mean recombination rate (y-Axis) per size category (top)

In contrast, particularly the dataset of ROHs identified using PLINK_B displayed more frequently wide areas in the genome not covered by ROHs. However, all three datasets of called ROHs either based on RZooRoH or PLINK showed a negative correlation between ROH length and the mean recombination rate resulting in a higher frequency of longer ROHs in regions with a tendency of low recombination rates (Fig. 9).

### Common ROHRs in heritage breeds

ROHRs detection of six pigs designated as heritage big breeds (Kune Kune, Duroc, Gloucester Old Spot, Iberian, Meishan, Yorkshire) revealed in total 22,396 shared ROHRs (PLINK_A) harbouring 3,583 genes, 15 ROHRs harbouring 10 genes (PLINK_B) and 15,924 ROHRs harbouring 4,341genes (RZooRoH, Table 2, Additional file 8: Table S8). In total, 1,861 of 3,583 genes detected in ROHRs using PLINK_A as well as all 10 genes identified in ROHRs using PLINK_B were also identified in ROHRs detected by RZooRoH. Merging of the ROHRs-datasets obtained by PLINK (PLINK_A, 2.09% genome coverage) and RZooRoH (5.70% genome coverage) to test for the total number of calls, resulted in 28,654 ROHRs, covering 6.72% of the genome.

**Table 2:** Number of ROH regions for each investigated potential selection event. For each phenotype, tool and parameter set, the number of ROHRs, the number of SNPs of all ROHRs and number of overlapping genes from the genome annotation are indicated. In addition, the total length of all ROHRs (in bp) and the coverage of the autosomal genome (in %) are provided

Phenotype	Tool + Parameter set	Number of ROHRs	Number of SNPs	Intersecting genes	ROHR length [bp]	Genome coverage [%]
Heritage breed	PLINK_A	22,396	974,847	3,583	47,244,400	2.09
Heritage breed	PLINK_B	15	1,235	10	71,087	0.00
Heritage breed	RZooRoH	15,924	1,760,741	4,341	129,231,106	5.70
Lop ears	PLINK_A	31,913	2,759,191	4,831	136,669,724	6.03
Lop ears	PLINK_B	2,007	299,821	582	15,562,455	0.69
Lop ears	RZooRoH	14,824	2,482,022	4,299	165,751,847	7.32
Prick ears	PLINK_A	9,839	328,033	1,684	15,768,865	0.70
Prick ears	PLINK_B	0	0	0	0	0.00
Prick ears	RZooRoH	5,812	604,787	1,630	41,990,018	1.85
Disease resistance	PLINK_A	13,427	621,064	2,417	31,092,255	1.37
Disease resistance	PLINK_B	279	43,728	78	2,261,875	0.10
Disease resistance	RZooRoH	11,774	883,853	2,969	58,704,190	2.59

**Fig. 10:**
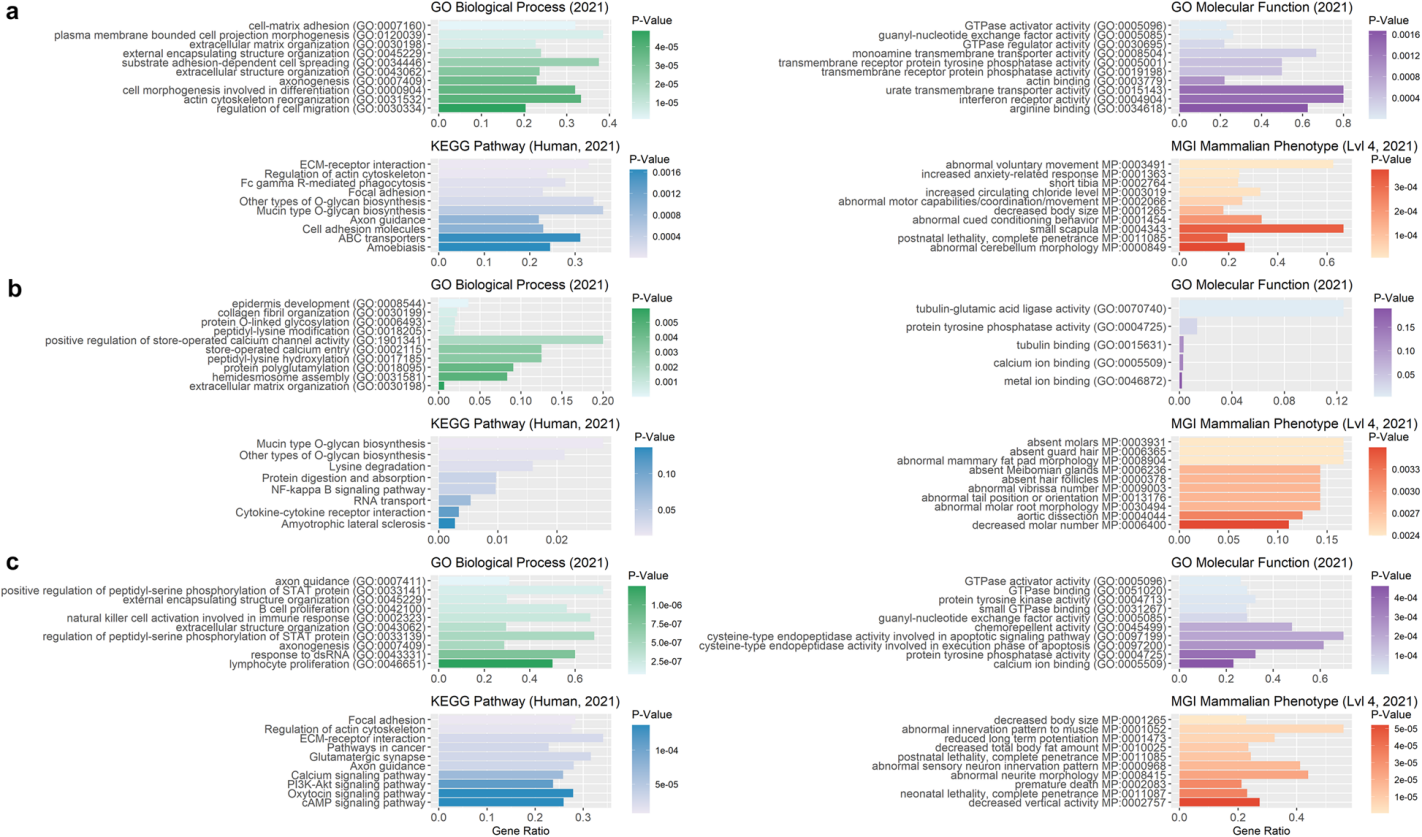
Functional enrichment analysis for genes overlapping with heritage-breed-associated ROHRs identified with PLINK and RZooRoH. Illustration of 10 highest enriched GO terms for enrichR databases “GO_Molecular_Function_2021”, “GO_Biological_Process_2021, “Human Phenotype Ontology”, “KEGG_2021_Human” and “MGI_Mammalian_Phenotype_Level_4_2021” sorted by *p*-value (x-axis), for all genes overlapping with ROHRs detected with either PLINK_A (**a**, 20 SNPs), PLINK_B (**b**, 120 SNPs) or RZooRoH (**c**), and shared by all individuals of heritage pig breeds

ROHRs obtained using PLINK_A revealed significantly enriched terms for biological processes such as regulation of platelet activation (GO:0,010,543), homophilic cell adhesion via plasma membrane adhesion molecules (GO:0,007,156) and sensory perception of sound (GO:0,007,605, Additional file 9: Table S9). For ROHRs from PLINK_B, no enrichment hits were found for pig genes in Panther database, wherase ROHRs detected using RZooRoH resulted in significantly enriched terms for biological processes such as locomotory behavior (GO:0,007,626), axon guidance (GO:0,007,411) and neuron projection guidance (GO:0,097,485). Moreover, gene set enrichment for 2,668 human orthologue genes out of 3,583 pig genes (PLINK_A), 8 human orthologous of 10 pig genes (PLINK_B) and 3,328 human orthologous of 4,341 pig genes (RZooRoH) revealed several significantly enriched terms (Fig. 10).

Furthermore, for PLINK_A, 1,398 of the 3,583 genes intersected with gene sets associated with the terms “anxiety or stress” in NCBI database (Additional file 10: Table S10). Further 816 genes intersected with the NCBI terms “immun* or “inflam*”, 17 genes with the “disease resistance” and 12 genes with “meat quality”. Moreover, three out of 10 genes overlapping with the ROHRs detected with PLINK_B, were found in “anxiety or stress”-related gene set. Considering ROHRs obtained from RZooRoH, 1,641 of 4,341 genes within ROHRs were found to overlap with gene sets associated with the terms “anxiety or stress”, 1,069 genes overlap with the NCBI terms “immun*” or “inflam*”, 20 genes with “disease resistance” and 20 genes with “meat quality”.

### Selection signatures for favourable genotype–phenotype effects

To identify potential signatures under selection for disease resistance as a trait of importance in pig breeding, we investigated common ROHRs in pig breeds (Angeln Saddleback × Mangalica, Minipig, Mini-Lewe, Goettingen Minipig, Meishan, Wuzhishan minipig) exclusively harbouring the disease resistance-associated genotype T/T (*GBP5*: g.127301202G > T) identified in *GBP5* [43]. We aimed at testing the hypothesis [44], that this associated variant might play a role in disease resistance across breeds.

In total, 13,427 ROHRs harbouring 2,417 genes (PLINK_A), 279 ROHRs harbouring 78 genes (PLINK_B) and 11,774 ROHRs harbouring 2,969 genes (RZooRoH) were detected (Table 2, Additional file 11: Table S11). In total, 1,236 of 2,417 genes intersected between the two ROHRs datasets obtained from PLINK_A and RZooRoH, all 78 genes overlapped between PLINK_B and RZooRoH, and 73 genes were identified both in ROHRs detected using PLINK_A and PLINK_B. However, for none of the ROHRs detected by rule- or by model-based approaches we could confirm an overlap with the region of the disease resistance-associated genotype itself (SCC4:127,301,202). In addition, further investigation of the total number of calls by merging the ROHRs-datasets obtained by PLINK (PLINK_A, 1.37% genome coverage) and RZooRoH (2.59% genome coverage), resulted in 20,128 ROHRs, covering 3.28% of the genome.

**Fig. 11:**
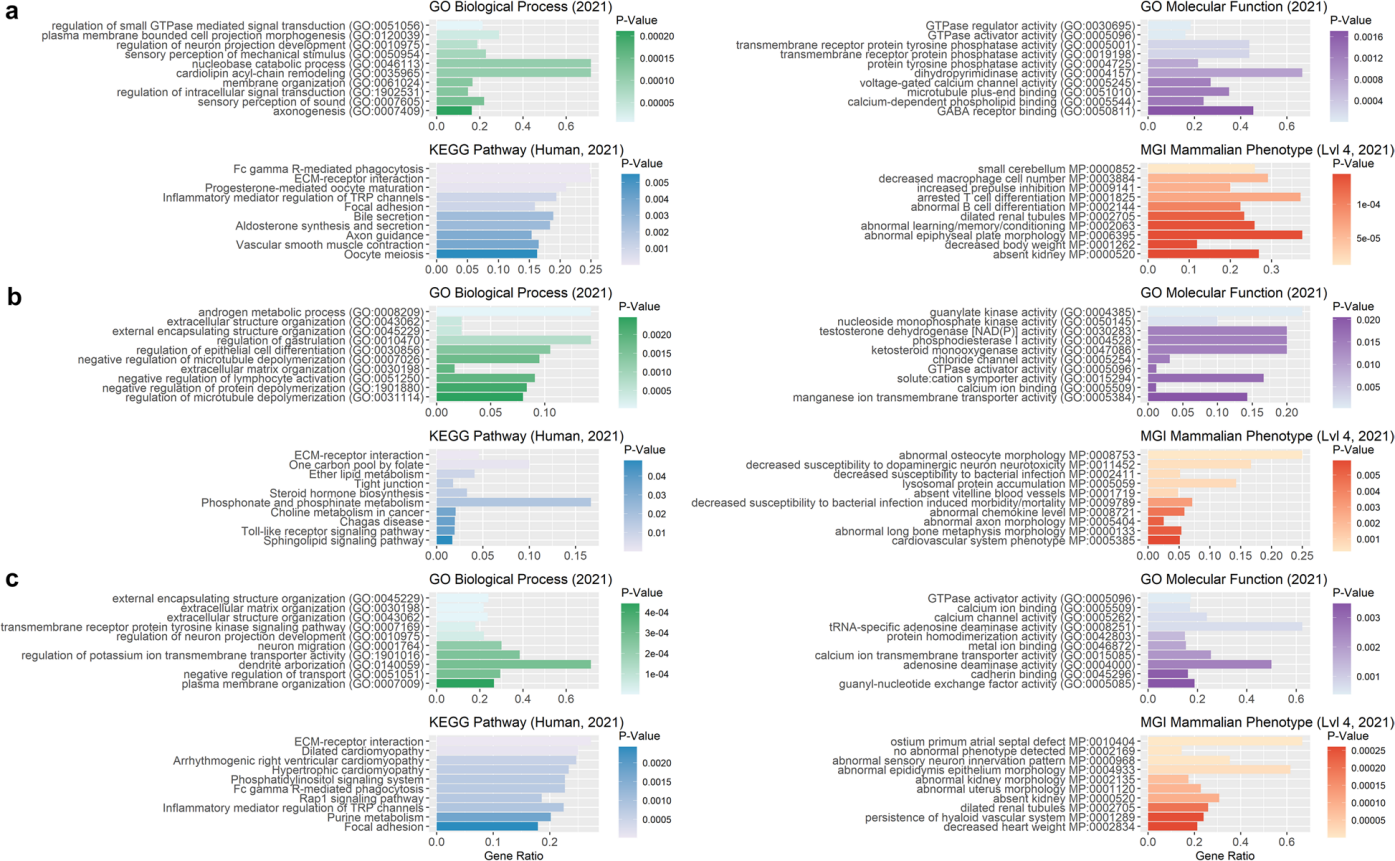
Functional enrichment analysis for genes overlapping with disease-resistance-associated ROHRs identified with PLINK and RZooRoH. Illustration of 10 highest enriched GO terms for enrichR databases “GO_Molecular_Function_2021”, “GO_Biological_Process_2021”, “KEGG_2021_Human” and “MGI_Mammalian_Phenotype_Level_4_2021” sorted by *p*-value (x-axis), for all genes overlapping with ROHRs detected with either PLINK_A (**a**, 20 SNPs), PLINK_B (**b**, 120 SNPs) or RZooRoH (**c**), and shared by all individuals with T/T genotype (GBP5: g.127301202G > T) associated with disease resistance

Nevertheless, genes within detected ROHRs (PLINK_A) resulted in significantly enriched terms for biological processes such as vesicle-mediated transport in synapse (GO:0,099,003), synaptic vesicle cycle (GO:0,099,504) and regulation of GTPase activity (GO:0,043,087) (Additional file 9: Table S9). Furthermore, we found several significantly enriched terms in gene set enrichment analysis for 1,791 human orthologue genes out of 2,417 pig genes (Fig. 11).

For PLINK_B, we did not find any significantly enrichment hits using the Panther data. However, 59 human orthologous of 78 pig genes revealed genes significantly enriched in negative regulation of T cell differentiation in thymus (GO:0,033,085), regulation of immature T cell proliferation in thymus (GO:0,033,084), and increased susceptibility to autoimmune diabetes. Furthermore, ROHRs obtained from RZooRoH revealed significantly enriched terms for biological processes such as cell-substrate adhesion (GO:0,031,589), cell–cell junction organization (GO:0,045,216) and regulation of transporter activity (GO:0,032,409). In addition, several significantly enriched termin for 2,232 human orthologue genes were identified.

Furthermore, intersections of two gene sets from the NCBI database with genes in ROHRs revealed 507 out of 2,417 genes (PLINK_A) overlapping with the genes associated to the terms “immun*” or inflam*” and ten genes related to the term “disease resistance” (Additional file 10: Table S10). Moreover, 16 of 78 genes (PLINK_B) as well as 620 of 2,969 genes (RZooRoH) in ROHRs intersected with genes assigned to the terms “immun*” or “inflam*”, as well as further 14 genes (RZooRoH) with “disease resistance”.

In addition to that, we investigated regions under potential selection for the ear types considered as an important breed defining feature. To identify homozygosity regions associated with lop ears as well as prick ears, filtering for ROHRs was performed using both PLINK parameter sets a and set b and RZooRoH. For prick ears, PLINK_A resulted in 9,839 shared ROHRs harbouring 1,684 genes and RZooRoH resulted in 5,812 shared ROHRs harbouring 1,630 genes, of which 658 genes were intersecting between the two results (Table 2). Using PLINK_B, no shared ROHRs were detected for the prick ear phenotype.

For all individuals with lop ears, PLINK_A resulted in 31,913 shared ROHRs harbouring 4,831 genes, PLINK_B in 2,007 ROHRs harbouring 582 genes, and RZooRoH in 14,824 ROHRs harbouring 4,299 genes. In total, 2,181 of these genes overlapped between the datasets detected using PLINK_A and RZooRoH, whereas 540 genes intersected between PLINK_A and PLINK_B.

Furthermore, merging of the total number of ROHR-calls for prick ear detected by PLINK (PLINK_A, 0.7% genome coverage) and RZooRoH (1.85% genome coverage), resulted in a total of 12,102 ROHRs, covering 2.25% of the genome. For lop ears, 36,688 ROHRs covering 10.42% of the genome were identified based on ROHRs-datasets obtained from PLINK (PLINK_A, 6.03% genome coverage) as well as RZooRoH (7.32% genome coverage).

**Fig. 12:**
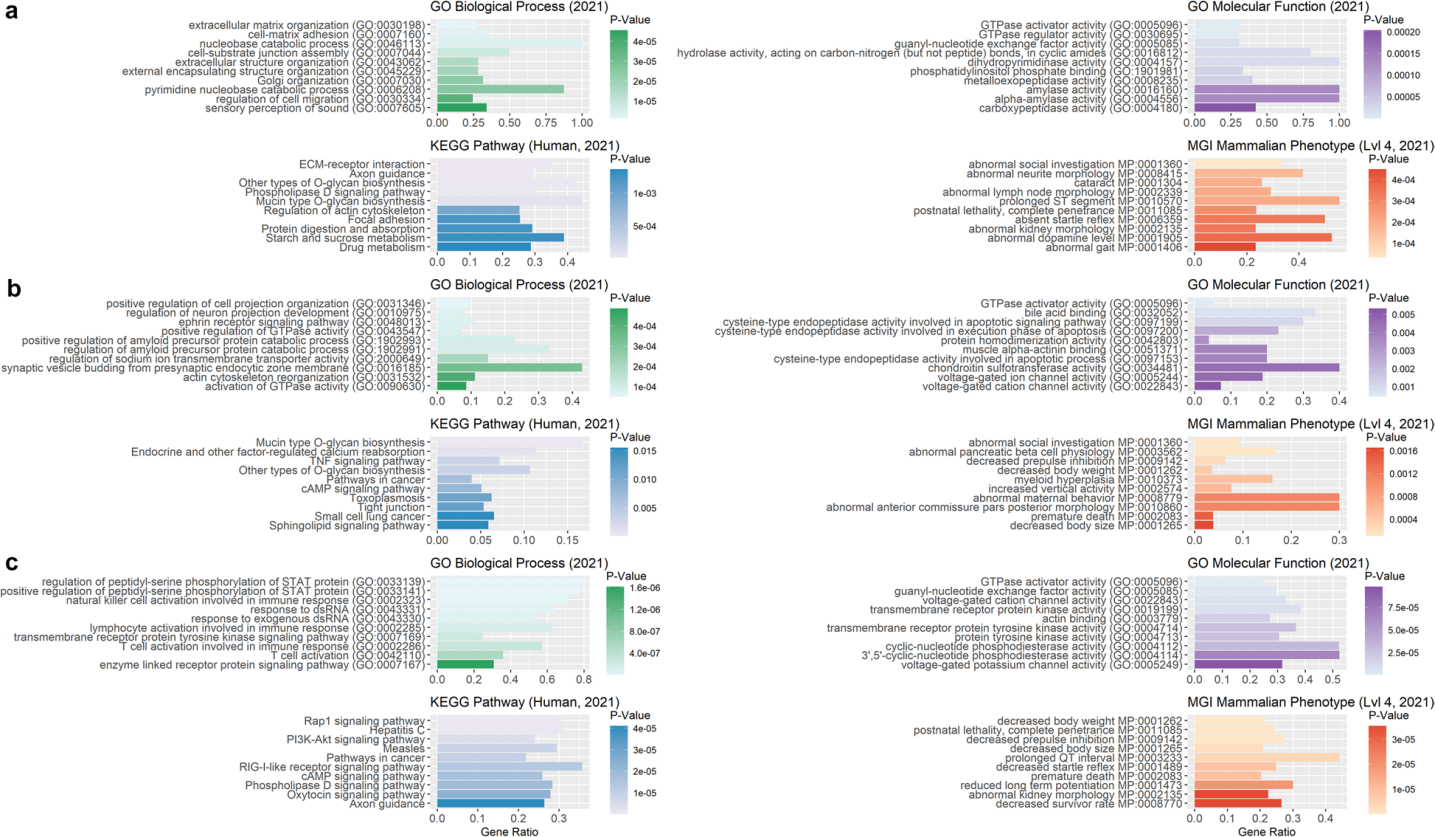
Functional enrichment analysis for genes overlapping with lop-ear-associated ROHRs identified with PLINK and RZooRoH. Illustration of 10 most significantly enriched GO terms for enrichR databases “GO_Molecular_Function_2021”, “GO_Biological_Process_2021”,”KEGG_2021_Human” and “MGI_Mammalian_Phenotype_Level_4_2021” sorted by *p*-value (x-axis), for all genes overlapping with ROHRs detected with either PLINK_A (**a**, 20 SNPs), PLINK_B (**b**, 120 SNPs) or RZooRoH (**c**), and shared by all individuals with lop ears

Enrichment analysis for lop ear-associated ROHRs based on PLINK_A, resulted in significantly enriched terms for biological processes including cell-substrate adhesion (GO:0,031,589), positive regulation of GTPase activity (GO:0,043,547), and regulation of GTPase activity (GO:0,043,087, Additional file 9: Table S9). Furthermore, gene set enrichment for 3,365 human orthologue genes out of 4,831 pig genes revealed several significantly enriched terms (Fig. 12).

Similar enrichment of genes could be found for lop-ear associated ROHRs called using PLINK_B, as well as for 424 human orthologous out of 582 pig genes in enrichR analysis. For RZooRoH we found significantly enriched terms for biological processes such as locomotory behavior (GO:0,007,626), regulation of neuron projection development (GO:0,010,975) and modulation of chemical synaptic transmission (GO:0,050,804) as well as significantly enriched terms in gene set enrichment analysis for 3,067 human orthologue genes.

**Fig. 13:**
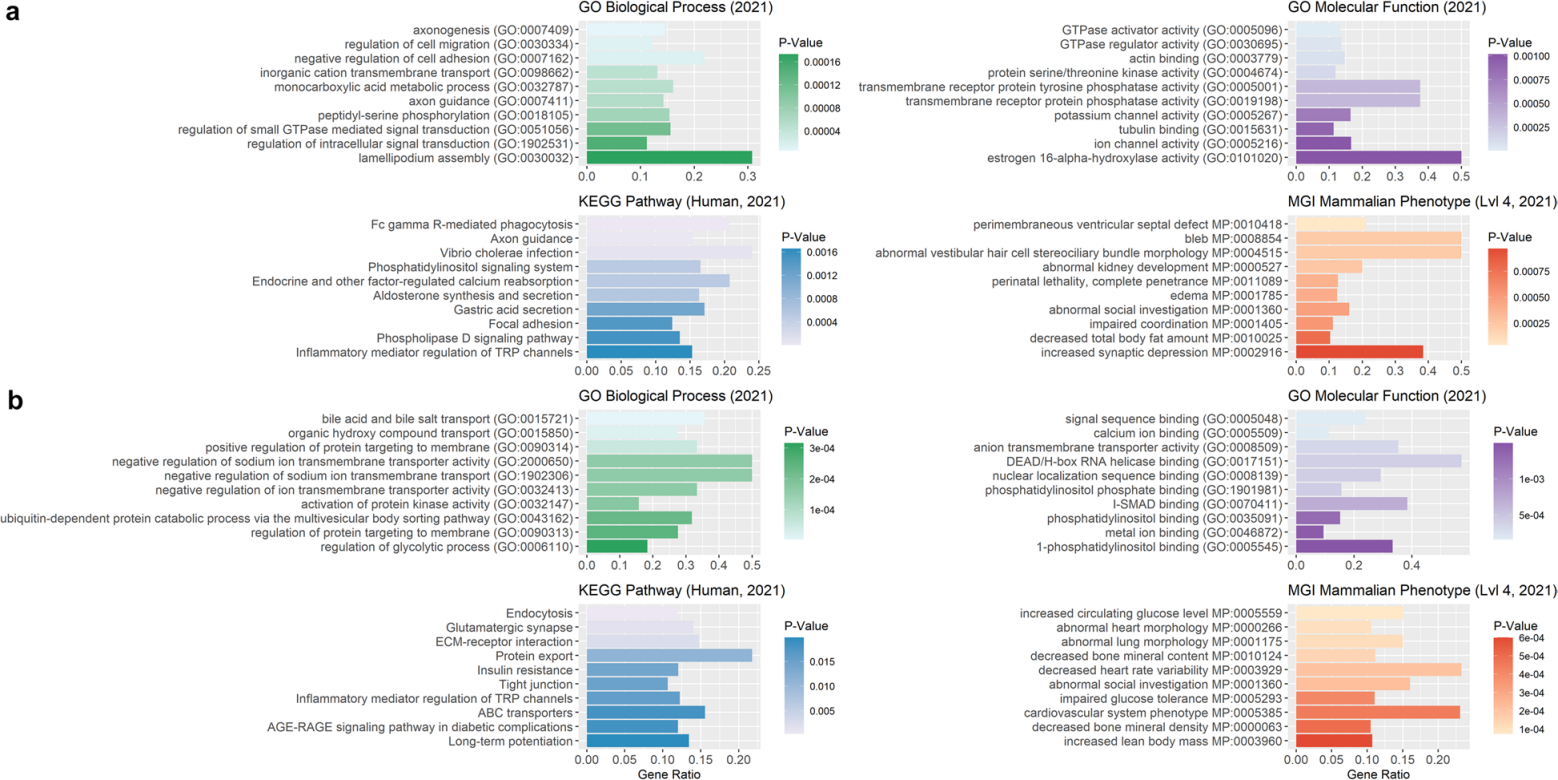
Functional enrichment analysis for genes overlapping with prick-ear-associated ROHRs identified with PLINK and RZooRoH. Illustration of 10 most significant enriched GO terms for enrichR databases “GO_Molecular_Function_2021”, “GO_Biological_Process_2021”, “KEGG_2021_Human” and “MGI_Mammalian_Phenotype_Level_4_2021” sorted by *p*-value (x-axis), for all genes overlapping with ROHRs detected with PLINK_A (**a**, 20 SNPs) or RZooRoH (**b**), and shared by all individuals with prick ears. For PLINK_B (120 SNPs), no ROHRs shared by all individuals with prick ears were detected

For the prick ear phenotype, common ROHRs could only be detected using PLINK_A and RZooRoH. For both methods, we found ROHRs harbouring significantly enriched pig genes and an enrichment of 1,303 human orthologous (1,684 pig genes, PLINK_A, Fig. 13) as well as 1,240 human orthologous (1,630 pig genes, RZooRoH).

For ROHRs detected by PLINK_A, we found lamellipodium organization (GO:0,097,581), lamellipodium assembly (GO:0,030,032) and homophilic cell adhesion via plasma membrane adhesion molecules (GO:0,007,156) to be signifcantly enriched among the biological processes (Additional file 10: Table S10). ROHRs obtained from RZooRoH were signifcantly enriched among the biological processes playing a role in regulation of cation transmembrane transport (GO:1,904,062), regulation of ion transmembrane transport (GO:0,034,765) and regulation of transmembrane transport (GO:0,034,762).

In addition, we found 3,076 of 4,831 genes (63%, PLINK_A), 404 of 582 genes (68%, PLINK_B) and 2,740 of 4,299 genes (64%, RZooRoH) identified in lop ear-associated ROHRs intersecting with NCBI seach term “ear”. For PLINK_A only one gene, namely *mitogen-activated protein kinase 3 (MAPK3)*, and for RZooRoH two genes *MAPK3* and *eyes absent homolog 1 (EYA1)* were identified in the overlap with the term list “outer ear” (Additional file 11: Table S11). For prick ear, 1,240 of 1,684 genes (74%, PLINK_A) and 1,152 of 1,630 genes (71%, RZooRoH) were found in the NCBI “ear” term list. No intersection could be found for term list “outer ear” neither for prick ear gene lists obtained with any tool nor for lop ear gene lists obtained from PLINK_B.

Moreover, we did not find any overlap of ROHRs with the pig ear-size associated QTL on chromosome 5 (SSC5) at 29.74–29.88 Mb containing *MSRB3* among other genes and no overlap with another QTL on chromosome 7 (SSC7) at 31.22–31.29 Mb containing the *PPARD* gene. However, in close proximity to SSC5 QTL we found five ROHRs in the dataset obtained by PLINK_A of which one contained the *glucosamine (N-acetyl)-6-sulfatase* (*GNS)* gene and one ROHRs in this area in the dataset from RZooRoH analysis (Additional file 12: Table S12). In three previously published highly differentiated regions on chromosome 5 and 7 [45] linked to ear morphology, we identified multiple overlaps with ROHRs detected by both tools. These overlapping ROHRs contain 35 candidate genes associated with ear morphology. Of these 35 candidate genes, seven are implicated in ear cartilage development, including *GNS, homer scaffold protein 2 (HOMER2), KH RNA binding domain containing, signal transduction associated 2 (KHDRBS2), ras-related protein rab-23 (RAB23), ras association domain family member 3 (RASSF3), TANK binding kinase 1 (TBK1)* and *WASP homolog associated with actin, golgi membranes and microtubules (WHAMM).*

Furthermore, filtering of all variants located within ROHRs obtained with PLINK for genotypes associated with ear type revealed one variant in intron 5 (intron variant, ENSSSCT00000031470.3/ENSSSCT00000018785.4, c.463 + 82G > C) and exon 6 (missense variant, ENSSSCT00000081093.1, c.542G > C) on chromosome 12 at 11,229,528 bp of *ATP binding cassette subfamily A member 6 (ABCA6)* gene with a moderate effect (SIFT score: tolerated, 0.12). Both were homozygous wild type (0/0) for all lop-eared individuals and overlapped with a ROHR shared by all lop-eared individuals (Husum Red Pied, Turopolje, Mangalitza, Bentheim Black Pied, Duroc, Gloucester Old Spot, Iberian) plus homozygous mutant (1/1) for prick-eared pigs. However, the variant in the *ABCA6* gene was also found as homozygous mutant in the lop-eared Meishan pig (1/1) and heterozygous for the prick-eared Yorkshire pig (0/1). The same variant as well as further missense variants (ENSSSCG00000048926: ENSSSCT00000066589.1, c.326G > A, ENSSSCT00000066589.1, c.80G > A; ENSSSCG00000046730: ENSSSCT00000084435.1, c.3366 T > G; ENSSSCG00000031845: ENSSSCT00000036965.1, c.106A > G, ENSSSCT00000081497.1, c.-1569-52033A > G, ENSSSCT00000036965.1, c.592G > A, ENSSSCT00000036965.1, c.592G > C) and intron variants ENSSSCG00000044445: ENSSSCT00000081497.1, c.-1569-51547G > A, ENSSSCT00000081497.1, c.-1569-51547G > C) were also identified using RZooRoH for lop ears.

## Discussion

In present study, we demonstrated a strategic ROH detection by using different approaches as well as systematic parameter testing in order to identify signatures of potential selection in the genome. Various research studies based on ROH analyses in domestic animals have been performed so far, but a lack of standardized criteria for quality control of underlying datasets, specific recommendations for WGS data and definition of ROHs has been observed [2, 24]. This issue was addressed by researchers, evaluating the performance of different autozygosity detection algorithms as well as elaborating necessary requirements for ROH calling in SNP array data in livestock and pets [22, 23]. In our study, we created a workflow specifically for WGS data, which require very specific configurations for robust homozygosity calling [10], in an exemplary dataset from pig samples of various populations/breeds.

For quality control, iterations of coverage and missing call parameters for raw SNP data revealed a high dependency of the sequenced samples with the genome read coverage. We found a maximum read depth of about two-times the average coverage of the sample with the highest coverage in the dataset to be an appropriate setting for variable genome sequencing data, similarly as previously suggested for SNP array data [23]. The high variation we observed in the number of SNPs when different minDP and maxDP were applied, possibly explains previous findings of a very high sensitivity of ROH detection to parameters or thresholds used for sequencing and pruning of SNPs [14]. Thus, adjusting the parameters dependent on genome coverage, number of samples and missing SNPs represents a feasible approach to follow current recommendation, which proposes to produce a more uniform SNP coverage to run a more independent ROH calling from variation in SNP density [23]. This filtering step was suggested for both methods, rule- and model-based, to be run prior to ROH detection using a tool such as PLINK [19, 21]. Producing a defined input file of high-quality SNPs provides a huge advantage for ROH studies as it enables comparable analyses across different methods.

Thus, the quality-controlled SNP set went into ROH calling using the sliding window approach of PLINK, as well as the HMM approach offered by RZooRoH. Evaluation of PLINK’s default ROH-defining values clearly confirmed the suggestion that they are not suitable for all SNP datasets, particularly not for those derived from WGS data, and must necessarily be tuned to the characteristics of the underlying data to receive meaningful comparable results [13, 22]. Especially the size of the genome covered with SNPs and the total number of SNPs were shown to be key parameters to define ROH. In addition, dependencies of applied ROH detection parameters played an essential role: Our iterations of PLINK’s scanning window parameters verified huge differences in numbers and sizes of ROH, particularly dependent on SNP density, the minimum length in kb, and the hit rate of all scanning windows containing a SNP. This underlined the usefulness of recently suggested formula modelling these dependencies to result in the best fitting PLINK parameter sets [7, 22, 25].

Furthermore, as previously requested by a ROH-parameter study in SNP array data [22], we investigated the role of *homozyg-gap,* defining the maximum interval between two homozygous SNPs, in WGS data. Similarly, as reported for the low to medium density SNP sets [22], we found only minimal deviations in length and number of ROH when adjusting gap settings and thus suggest the default of 1000 kb as an appropriate value for high density SNP sets. Just as well, our data confirmed the assumption that in PLINK heterozygous calls per window should be tolerated to a certain extent, particularly if (a) high density SNP sets are used and (b) domestic animal populations are investigated, which can be expected to have much higher levels of autozygosity in comparison to human genomes [6, 22]. In some cases, this might result in a merge of two homozygous segments [22], but represents a reasonable trade-off to allow more accurate long ROH detection plus an increased sensitivity for shorter ROH calls as well.

In particular, the accurate detection of shorter ROHs was shown to be challenging, which requires a high number of reliable SNPs as well as more stringent ROH calling parameters adapted to the high SNP density [2, 13, 14]. Our findings revealed that shorter ROHs can only be detected using PLINK with significantly lower *homozyg-snp* and *homozyg-window-snp* values, in comparison to the computed settings formula-based as discussed above. Thus, we found scaling down the calculated value by 80–85% and adjusting the dependent parameters (PLINK_A), accordingly, increased the hit rate for shorter ROHs significantly. These adjustments appear to be necessary when using PLINK to cover the high number of smaller ROHs, which were found to be present in high frequency in domestic animals [7, 10, 14]. Larger ROHs were also detected reliably using these settings (PLINK_A) as well as the formula-based settings (PLINK_B). However, we suspect the results from PLINK_B tend to a higher number of false negative calls.

In contrast to the above-mentioned parameter tests necessary to apply suitable settings for a rule-based ROH calling, model-based approaches such as RZooRoH offer an ad hoc procedure to define optimal window sizes and thus do not require prior definition of ROH parameters [19, 46]. Subsequently, we could confirm the model-based approach offered by RZooRoH to be a user-friendly procedure [19] with only few parameter adjustments necessary. Nevertheless, the assignment of HBD segments into different classes has to be regarded with care and was suggested to be run based on a selection of pre-defined HBD classes [19]. Based on this estimation, we limited in our experiment the number of classes by defining a maximum rate R_K_ of 10,000 analogous to the time of domestication of the pig [47], because otherwise we would have called extremely small HBD segments resulting in a potential increase of false positive ROH detection rates. Subsequently, our results confirmed previous suggestion that PLINK is more stringent with regard to ROH size than a model-based approach as a minimal ROH length has to be defined prior to the run [22]. However, as long as the weaknesses of the different methods were taken into account and the parameters adjusted accordingly, the differences between the approaches PLINK_A and RZooRoH were small (overlap > 50% from both sides), similar as it was previously suggested [27], particularly with regard to ROHs ≤ 20 kb. However, with respect to the total number of detected ROHs, the results from RZooRoH showed a more balanced size distribution of called ROHs across all chromosomes and individuals. We can only suggest that this comparatively high total number of ROHs detected by RZooRoH might be either a result of a better fitting model producing lower false negative rates compared to rule-based methods [19, 26] or might display a number of false positive ROH calls. However, independent from the used method or parameter set, we were able to confirm the suggestion that longer and probably younger ROHs tend to occur in low-recombination regions [46].

Furthermore, as expected, the number and size of ROHs varied widely among pigs with a history of greater inbreeding events in recent times, like Yorkshire pigs [28] in contrast to less selected populations or hybrids. These findings support the assumption that ROHs are important determinants of recent and more ancient population bottlenecks and inbreeding events [8]. It was postulated that longer ROHs are more likely to be neutral and degraded by recombination, whereas small ROH are retained and more often shared among individuals [6, 8]. According to our estimations, the detected ROHs in the small ROH length categories might go back in history for more than 2000 years, taking into account the possible deviations of the assessed time span affected by differences in generation intervals across pig breeds [41]. This is intriguing, as ROHs are proposed to highlight selection footprints in the genome, potentially harbouring genes or non-coding functional elements and mutations associated with economically important phenotypic traits [8, 48].

Thus, we demonstrated the applicability of ROHs analyses to track down regions under potential selection and thereby narrow down potential candidate genes in our pig dataset. Compared to PLINK_B, the number of common ROHRs in the investigated phenotype groups was higher when PLINK_A or RZooRoH was applied, allowing detection of smaller ROHs.

This was particularly noticeable in the group of heritage pig breeds, designated as rare breeds offering unique genes imparting valuable traits such as disease resistance or effective forage utilization [49], but with less resilience to external stressors as observed in high production pigs [50]. The selection focus on fitness-related traits in heritage pig breeds was underlined by various genes in potential signatures of selection that might be involved either in supporting disease resistance and/or disease tolerance, the adaptive ability in preserving homeostasis without affecting the pathogen per se [51]. We propose that the latter probably is the most prominent characteristic under selection in heritage pig breeds. This assumption was supported by screening the disease resistance-associated T/T genotype (rs340943904) in *GBP5*, which was postulated to affect the response against PRRS infection [43] and was only present in one of the studied heritage pig breeds (Meishan) but in five other investigated populations, mainly minipigs. This might be a result from selection for an improved disease resistance of miniature pigs used as a model organism for biomedical research [52]. Notably, we could not identify an overlap with a ROHR in all pigs harbouring the favourable T-allele but instead detected different ROHs possibly accounting for the effective inflammasome-assembly in these pigs. This finding substantiates the accuracy of our ROHR analysis, as the low frequency of the favorable allele, as reported in a segregation-study in 20 European local breeds [44], was raising the expectation that indeed no selection signature at this specific locus is present.

As a result, our findings are consistent with the idea that main selection criteria for pigs, often related to performance, health and morphological traits [44], lead to footprints in the genome that can be identified as long stretches of homozygous genotypes. In particular, the morphologic traits are prioritized by breeders as special characteristics and are therefore breed defining [44]. Exemplary as such specific trait under selection, we examined ear shape in pigs. Our data were not only able to support previously identified signatures of diversifying selection associated with ear morphology [45], but also provided new candidate genes potentially playing a role for ear shape in pigs. *MAPK3* was discovered as a particularly interesting candidate gene for outer ear development of lop eared pigs, known to play an essential role in the *MAPK/ERK* cascade, which mediates various biological processes such as cell growth, adhesion, survival and differentiation by regulating transcription, translation and rearrangements of the cytoskeleton [53]. Furthermore, *MAPK* was shown to be an important protagonist in chondrocyte differentiation and cartilage tissue formation processes [54, 55]. It was found to be involved in a signalling cascade initiated by *KIT* (also located in lop-ear associated ROHR) and therefore might support the formation of ear cartilage tissue as well. Furthermore, *Eya1* was identified using RZooRoH as another interesting candidate for outer ear development in lop eared pigs. *Eya1* plays a role in murine ear development [56] and is associated with Branchio-Oto-Renal (BOR) syndrome in humans, an autosomal dominant early developmental defect characterised by varying combinations of branchial, outer, middle and inner ear, and renal anomalies [57].

In addition, to that, ROH analyses using PLINK/RZooRoH revealed a potential candidate missense mutation in the *ABCA6* gene within a ROHR homozygous mutant exclusively (except for Meishan pig) in prick eared pigs. ABCA6 might be involved in ear development, although the function of this ABC transporter is not clear yet [58]. Furthermore, the role of the mutant allele in Meishan pigs probably needs further exploration in the future. Meishan develop extraordinarily large and floppy ears, which might be genetically determined by a different mutation, similar as it was reported for the characteristic ear phenotype in Chinese Erhualian pigs [59]. These results show that different interesting candidate genes could be detected in ROHRs, which were either based on ROHs identified using PLINK, RZooRoH or both. This leads to the assumption that, although RZooRoH is apparently detecting a higher number of ROHs, some ROHRs might be missed by this approach. For this reason, we suggest for those studies, which are primarily searching for genomic regions under selection for specific phenotypes and/or causative variants and provide a genotype-filtering in a second step to reduce the number of false positive calls, running a simultaneous ROH detection using a rule- and a model-based approach and performing our suggested optional merging step for ROHRs might be the best way to avoid missing genes of interest.

## Conclusions

In our study, we present a workflow for ROH detection using both a rule- and a model-based approach. We underlined the important role of high-quality SNP datasets as prerequisite for ROH calling in WGS data. The results from our exemplary pig dataset of various populations/breeds demonstrated the limits of parameter estimations exclusively based on formula to define ROHs, particularly with regard to the rule-based detection targeting shorter ROHs. Subsequent comparison of ROH calling approaches demonstrated the high efficiency of both rule- and a model-based method for ROH detection if properly applied and underlined their importance with regard to the identification of candidate genes. We were able to identify potential footprints of selection events taking place to some extent far back in the past defining pig breeds or populations and reflecting their characteristics and favoured phenotypes. These data suggest that ROH detection, if based on a systematic dataset-adjusted approach, is an efficient way to open up a window into the genome finding traces of selection.

## Methods

### Samples and whole genome sequencing

In total, 16 samples from different pig breeds/populations and four crossbreeds were used for this analysis based on WGS data (Additional file 13: Table S13). Data files of 10 pig samples were derived from the NCBI Sequence Read Archive (SRA). Genomic DNA of further 10 animals were isolated from either EDTA-blood, hair roots, skin or muscle tissue using an in-house chloroform extraction protocol [60]. In total, 200 ng DNA of each sample was sonicated with a Covaris S2 system (Covaris, Woburn, Massachusetts, USA) using the following settings: 10% duty cycle, intensity 5, 40 s. Library preparation was performed using the KAPA HyperPrep Kit according to the manufacturer’s guidelines (Hoffmann-La Roche, Basel, Switzerland). Adapters from the NEXTFlex UDI set B (PerkinElmer, Waltham, Massachusetts, USA) were used for multiplexing, followed by a 0.6X-0.8X double-sided bead size selection and four cycles of PCR. The quality of the libraries was estimated on an Agilent 2100 Bioanalyzer using the Agilent High Sensitivity DNA Kit (Agilent Technologies, Santa Clara, California, USA). Subsequently, the libraries were sequenced paired end for 150 bp on an Illumina NovaSeq 6000 (Illumina, San Diego, California, USA). All animal experiments were conducted according to the national and international guidelines and approved by animal ethics committee of the Lower Saxony state veterinary office Landesamt für Verbraucherschutz und Lebensmittelsicherheit, Oldenburg, Germany (registered at 33.9–42,502-05-17A217).

### Whole-genome sequence analysis

All fastq-files were quality controlled using FastQC, version 0.11.8 [61] and underwent indexing with Picard tools [62]. Adapter trimming and low complexity filters were applied using the FASTQ pre-processor fastp, version 0.20.0 [63], with the following settings: detect_adapter_for_pe, -low_complexity_filter, -complexity_threshold 1, -cut_front -cut_front_window_size 1 -cut_front_mean_quality 20 -cut_tail -cut_tail_window_size 1 -cut_tail_mean_quality 20 -qualified_quality_phred 15 -unqualified_percent_limit 70 -n_base_limit 50 -average_qual 0 -disable_length_filtering -disable_trim_poly_g. Finally, all files were mapped to the reference genome Sscrofa11.1 (accessed from ENSEMBL, release 101) using the Burrows-Wheeler Alignment tool (BWA), version 0.7.17-r1188 [64]. Variants were called using GATK tools, version 4.1.9.0, [62] Base Recalibrator, Haplotype Caller, Base Quality Score Recalibrator and Calculate Genotype Posteriors and underwent variant effect prediction using SNPEff, version 4.3t, build 2017–11-24 [65].

### Evaluation of SNP filtering conditions

After variant calling, all variants on chromosomes MT, X, Y, and all scaffolds were excluded using vcftools, version 0.1.15 [66]. In addition, all INDELs and sites with less or more than two alleles, a minor allele count less than one and a base quality score of less than 30 were removed *(–min-alleles 2 –max-alleles 2 –remove-indels –mac 1 –minQ 30*). Then, four different filtering parameters of vcftools were tested in two different test settings in order to estimate the quality and number of SNPs as outcome (see Table 3). In the first test setting, SNPs were filtered for different minimum (*minDP*) and maximum read depths (*maxDP*). For the visual inspection, the number of SNPs was plotted for each tested parameter combination. The setting with the best outcome (*minDP 6, maxDP 95*) from the first test setting was then used to produce a variant set for the second test setting. In the second test setting, SNPs were further filtered for different maximum numbers of allowed missing genotypes (*max-missing-count*) and minimum mean read depths (*min-meanDP*) over all individuals. Based on the second test setting, the high-quality variant set obtained from analysis with max-missing-count 15 was used for all subsequent ROH analyses. SNPs were not pruned for linkage equilibrium or minor allele frequency prior to ROH detection, according to previous recommendation [22].

**Table 3:** Overview of parameters evaluated for filtering SNPs. Vcftools test settings and subsequent ranges of investigated specifications are displayed

Test setting	Parameter	Tested range	Description
1	minDP	2–16 (interval 2)	minimum read depth—allow only SNPs with given minimum read depth
1	max DP	30–100 (interval 5)	maximum read depth—allow only SNPs with given maximum read depth
2	max-missing-count	8–18 (interval 2)	maximum number of allowed missing genotypes—exclude SNPs with more than defined number of missing genotypes over all individuals
2	min-mean-DP	10–30 (interval 2)	minimum mean read depths—allow only SNPs with at least the given mean read depth values over all individuals

### Rule-based approach

The rule-based ROH detection tool PLINK, version 1.90b6.21 [67], was used to call ROHs using a sliding-window approach. As this method requires an optimization for every dataset, eight different ROH detection parameters were tested in four different test settings (Table 4). For this purpose, custom parameters were calculated according to previously suggested formula. Then, ROH detection was performed for these calculated values as well as for values in a higher or lower range within defined intervals, to test for most effective ROH calling settings. All results were compared to their default value according to PLINK.

**Table 4:** Overview of parameters evaluated for the detection of ROHs using PLINK

Test setting	Parameter	Tested range	Description
1	*homozyg-window-snp*	20—150 (interval 10)	scanning window size—number of SNPs a scanning window contains
1	*homozyg-window-threshold*	based on homozyg-window-snp	scanning window threshold—proportion of overlapping windows that must be homozygous to define a given SNP as part of a homozygous segment
2 + 3	*homozyg-snp*	20—150 (interval 10)	min. number of SNPs per ROH—minimal number of SNPs per ROH
2 + 3	*homozyg-kb*	based on homozyg-snp	min. ROH length [kb]—minimal desired length of a ROH in kb
2 + 3	*homozyg-density*	0.04–0.2 (interval 0.04)	min. inverse density [kb/SNP]—on average a ROH must have at least 1 SNP per the defined number of kb
2 + 3	*homozyg-gap*	50, 100, 250, 500, 1000	max internal gap [kb]—max interval below that two SNPs are considered adjacent. If two SNPs within a segment are too far apart, the segment is split
4	*homozyg-window-het*	0,1,2,3	max. number of heterozygous SNPs per window
4	*homozyg-window-missing*	3,5,7	max. number of missing SNPs per window

In the first test setting, the impact of two parameters affecting the characteristics of the scanning window used for ROH detection was evaluated. We assessed the scanning window size (*homozyg-window-snp*, default: 50) and scanning window threshold (*homozyg-window-threshold*, default: 0.05). All other parameters either were set to default (default test set) or were set based on our calculations (custom test set). The parameter *homozyg-window-snp* was calculated considering three factors; 5% false positive ROHs, a total of 32,664,930 SNPs and a mean percentage of 15.6% heterozygous sites in our data set based on a previously suggested modified formula [7, 25]:

$$homozyg-window-snp=\frac{{\mathrm{log}}_{e}(\frac{\alpha }{{n}_{s} *{n}_{i}})}{{\mathrm{log}}_{e}(1-het)}=\frac{{\mathrm{log}}_{e}(\frac{0.05}{32664930})}{{\mathrm{log}}_{e}(1-0.156)}=119.677\approx 120$$

with n_s_ the number of SNPs per individual, n_i_ the number of individuals, α the percentage of false positive ROH and *het* the mean heterozygosity across all SNPs. The scanning window threshold was calculated dependent on the scanning window size based on previously suggested formula [22]:

$$homozyg-window-threshold=floor\left(\frac{{N}_{out}+1}{L}, 3\right)=floor\left(\frac{4+1}{119.667}, 3\right)=0.04$$

with N_out_ the desired number of final outer SNPs on either side of the homozygous segment that should not be included in the final ROH, L the scanning window size and ‘, 3’ indicating flooring with three decimals.

In the next step, the effect of four different parameters defining the characteristics of a potential ROH segment was evaluated. Namely, the minimum number of SNPs per ROH (*homozyg-snp*, default: 100), the minimal length of a ROH in kb *(homozyg-kb*, default: 1000), the minimal inverse density of SNPs per kb a ROH must have (*homozyg-density*, default: 50) and the maximal gap between two SNPs in a ROH segment in kb (*homozyg-gap*, default: 1000). In our custom test setting, defined ranges of custom values for *homozyg-snp*, *homozyg-kb* and *homozyg-gap* were tested. All other parameters were set either to custom (calculations based on formula) or default values, including *homozyg-kb*. In the third test setting, defined ranges of custom values for *homozyg-density, homozyg-kb* and *homozyg-gap* were evaluated. Similar to the second test setting, all other parameters were set to either custom or default values, including *homozyg-snp*. The minimum number of SNPs per ROH, *homozyg-snp*, was calculated based on the formula described above for the scanning window size *(homozyg-window-snp)* [2, 25]. Furthermore, the minimal inverse density, *homozyg-density*, was calculated by dividing the total genome size covered by SNPs (2,265,774.640 kb) by the total of 32,664,930 SNPs covering the genome. Hence, the minimal length of a ROH, *homozyg-kb*, was calculated as product of *homozyg-density multiplied by homozyg-snp*.

Based on the first three tests, two custom parameter sets, one set considered to be the most effective for detection of shorter ROHs comprising at least 20 homozygous SNPs in a window (PLINK_A), and one set determined according to the calculated values comprising a minimum of 120 homozygous SNPs in a window (PLINK_B) were defined. For both parameter sets, the impact of the maximal number of heterozygous (*homozyg-window-het*, default: 1) and missing SNPs allowed per scanning window (*homozyg-window-missing*, default: 5) were evaluated in a further test setting. For both PLINK_A and PLINK_B, *homozyg-window-het* was tested for 0–3 SNPs and *homozyg-window-missing* for 3, 5 and 7 SNPs admitted per window. After considering the default settings for both window parameters to be most effective, the optimized settings “PLINK_A and PLINK_B” were applied for final ROH calling and the obtained results were used further analyses.

### Model-based approach

In addition to the rule-based method, a model-based approach using an HMM was applied by the tool RZooRoH, version 0.3.1 [19, 27]. In a first step, a KR model without any predefined states was implemented to estimate the optimal R_K_ rates for each ROH class over all individuals. A dependency of R_K_ rates with the length of ROH segments can be observed: the expected length of HBD segments is equal to 1/R_K_ in Morgan. R_K_ is the rate of the class k corresponding to ancestors present approximately 0.5 × R_K_ generations ago [68]. Thus, the rates R_K_ for the mixKR model were calculated as the median of all rates R_K_ estimated for each individual in the respective ROH class by the KR model. Assuming an average generation interval (GI) of two for pigs [41, 42] and a domestication history of 10,000 years [47], a maximum rate R_K_ of 10,000 was set. Finally, based on these parameter estimations, a mixKR model with 8 classes and R_K_ rates equal to 20, 29, 72, 239, 740, 838, 4,242 and 10,000, as well as a genotyping error rate of 0.25% as suggested by Ferenčaković et al. [6] was run for all 20 pigs. To assign each SNP position to the positions of HBD or non-HBD, the Viterbi algorithm was run as default [69].

### Size distribution of ROHs

ROHs called using the two PLINK parameter sets a and b as well as RZooRoH were investigated for reciprocal intersection of detected ROHs using the function intersect in bedtools, version 2.29.2 [70], allocated into size categories of “0–20 kb”, “20–50 kb”, “50–250 kb”, “250–500 kb” and “ > 500 kb” and plotted using the R package ggplot2 [71]. Given the approximate correlation between ROH length and the recombination distance from the common ancestor over time [23], the approximate age of the underlying inbreeding event was calculated for all lengths (L) in Mb assuming that 1 cM corresponds to 1 Mb and an average GI of two [41, 42] according to the following formula [72]:

$$Age = \frac{100}{2L}\times \mathrm{ GI}$$

To examine the distribution of ROH lengths across all individuals for all tools, we calculated the average recombination rate for all ROHs based on sex-averaged map of the landscape of pig recombination rate in 1-Mb windows obtained from Johnson et al. [73] and examined the distribution of ROHs across different size categories as a function of recombination rate.

### Identification of ROH regions and functional enrichment analysis

ROHR calling was performed based on the identified ROHs obtained from PLINK_A and PLINK_B as well as RZooRoH, using a custom script in R, v. 4.1. For this purpose, all SNPs located within ROHs were obtained for each sample individually, merged across individuals, and searched for overlaps within assigned groups (phenotype of interest), which were then designated as ROHRs. Finally, our ROH detection approach was verified by investigating the obtained data for specific research questions on particular selection events. First of all, we identified ROHRs for each sequenced pig obtained from different breeds/populations as well as for a cluster of pigs common as purebred pigs with a long-established breeding history and endangered status, so called heritage pig breeds (see also Additional file 13: Table S13). Then, to address favourable genotype–phenotype effects, common ROHRs were identified for a group of pigs with a higher disease resistance probability based on their T/T genotype (g*uanylate binding protein 5 (GBP5)*: g.127301202G > T; rs340943904) located on SCA4:127,301,202 [43, 44]. This genotype is associated with inflammasome-assembly during immune response and improved response to porcine respiratory and reproductive syndrome virus infection [43]. Furthermore, common ROHRs were examined for different ear types, a group of lop and a group of prick eared pigs. For this analysis, only pigs with clear phenotypes were used: for lop ears 7 individuals and for prick ears 8 individuals. Data from the Chinese Meishan pig known for its extremely large and floppy ears in the lop ear group were not considered, as this was confirmed to be an exclusive ear phenotype in this particular breed [59]. In addition, ROHRs obtained by PLINK (PLINK_A) and RZooRoH datasets were merged using bedtools merge function. More precisely, bed-files were concatenated, sorted by chromosome and position and merged using bedtools. By this approach, we investigated an optional merging step of the most efficient two approaches offering a total of ROHR calls obtained either from one of the tools or both in one file aiming at increasing the hit rate for those research questions targeting specific variants of interest, provided further genotype-based filtering is done in subsequent step.

Genes intersecting with the detected ROHRs were identified using bedtools with the current gene set from ENSEMBL release 104. Human orthologous genes were identified using g:Profiler, version e104_eg51_p15_3922dba [74]. Functional enrichment analysis was performed using PANTHER version 16.0 [75] with the databases “GO_Molecular_Function_2021” and “GO_Biological_Process_2021” [76, 77] for *Sus scrofa* gene set. In addition, we used enrichR, version 3.0 [78, 79] based on human orthologue genes using the databases “GO_Molecular_Function_2021”, “GO_Biological_Process_2021” [76, 77], “KEGG_2021_Human” [80–82] and “MGI_Mammalian_Phenotype_Level_4_2021” [83]. Human orthologues of genes associated with heritage breed ROHRs were interesected with four NCBI gene sets obtained by the search terms “anxiety or stress”, “disease resistance”, “meat quality” and “immun*” or “inflam*”. For the disease resistance, functional enrichment results were scanned for terms linked to this phenotype (“B cell”,”T cell”,”immun”,”inflam”,”interferon”,”macrophage”, “phagocytosis”) and human orthologous were interesected with two NCBI gene sets containing genes associated with the search terms “disease resistance” or the terms “immun*” or “inflam*”. In addition, gene sets related to the terms “outer ear” (comprising 15 genes) and “ear” (comprising 55,291 genes) were downloaded from the NCBI RefSeq database and intersected with the human orthologous of genes overlapping with the ROHRs associated to lop or prick ears. In addition, ROHRs were checked for overlap with a quantitative trait locus (QTL) on chromosome 5 at 29.74–29.88 Mb harbouring the *methionine dulfoxide reductase B3 (MSRB3)* gene associated with ear size and morphology in pigs [84–86] as well as another QTL on chromosome 7 at 31.22–31.29 Mb harbouring the *peroxisome proliferator activated receptor delta (PPARD)* gene linked to external ear morphology and fat deposition in pigs [59, 84, 87, 88]. Each QTL plus 600 kb up- and downstream of this region was examined for possible intersections with ROHRs detected for each phenotype. Moreover, all ROHRs were checked for potential overlaps with three previously published highly differentiated regions on chromosome 5 and 7 under strong diversifying selection between breeds with lop and prick ears [45].

## Supplementary Information

**Additional file 1: Table S1. **Iterations for different read depth parameters.

**Additional file 2: Table S2. **Filtering for mean read depth and missing genotypes.

**Additional file 3: Table S3.** Number of ROHs detected with PLINK for different scanning window parameters.

**Additional file 4: Table S4.** Number of ROHs detected using PLINK parameters defining a ROH segment.

**Additional file 5: Table S5.** Number of ROHs for tolerated heterozygous and missing SNPs in PLINK.

**Additional file 6: Table S6.** Number of ROHs overlapping between RZooRoH, PLINK_A and PLINK_B.

**Additional file 7: Table S7.** Overlap of ROHs between RZooRoH, PLINK_A and PLINK_B datasets in base pairs (bp).

**Additional file 8: Table S8.** ROH regions identified for heritage pigs using PLINK_A and PLINK_B and RZooRoH.

**Additional file 9: Table S9.** Functional PANTHER gene enrichment analysis for annotated pig genes identified in ROHRs.

**Additional file 10: Table S10.** Intersection of human orthologues genes detected in ROHRs with NCBI gene lists for phenotype-related terms.

**Additional file 11: Table S11.** ROH regions identified for disease resistance and ear phenotypes using PLINK_A, PLINK_B and RZooRoH.

**Additional file 12: Table S12.** Overlap of ROH regions (ROHRs) identified for lop and prick ear phenotypes.

**Additional file 13: Table S13.** DNA samples used for the evaluation of SNP filtering and ROH detection parameters.

## Data Availability

All WGS data are available at Sequence Read Archive (PRJNA795885, https://www.ncbi.nlm.nih.gov/bioproject/PRJNA795885).

## Abbreviations

ABCA6: ATP binding cassette subfamily A member 6
BWA: Burrows-Wheeler Alignment
CNVs: Copy number variants
EYA1: Eyes Absent Homolog 1
GBP5: Guanylate binding protein 5
GI: Generation interval
GNS: Glucosamine (N-acetyl)-6-sulfatase
HBD: Homozygosity-by-decent
HMM: Hidden Markov model
HOMER2: Homer scaffold protein 2
INDELs: Insertions or deletions
KHDRBS2: KH RNA binding domain containing, signal transduction associated 2
LD: Linkage disequilibrium
MAPK3: Mitogen-activated protein kinase 3
MSRB3: Methionine dulfoxide reductase B3
PPARD: Peroxisome proliferator activated receptor delta
QTL: Quantitative trait locus
RAB23: Ras-related protein Rab-23
RASSF3: Ras association domain family member 3
ROHRs: ROH regions
ROHs: Runs of homozygosity
SNPs: Single nucleotide polymorphisms
SRA: Sequence Read Archive
TBK1: TANK binding kinase 1
WGS: Whole genome sequencing
WHAMM: WASP homolog associated with actin, golgi membranes and microtubules
