# Supplementary material for: Simultaneous testing of rule- and model-based approaches for runs of homozygosity detection opens up a window into genomic footprints of selection in pigs
Source: BMC Genomics. 2022 Aug 6;23:564. doi: 10.1186/s12864-022-08801-4 (PMC9357325; doi:10.1186/s12864-022-08801-4)
Supplement: Supplementary file 1 — Additional file 1: Table S1. Iterations for different read depth parameters. [file 12864_2022_8801_MOESM1_ESM.docx]

Table S1. Iterations for different read depth parameters. The number of SNPs after filtering with minimum (minDP) and maximum read depths (maxDP) is displayed.

|  |  | **minDP** | | | | | | | | |
| --- | --- | --- | --- | --- | --- | --- | --- | --- | --- | --- |
|  |  | **2** | **4** | **6** | **8** | **10** | **12** | **14** | **16** |  |
| **max DP** | **30** | 32933744 | 32709374 | 32277489 | 31189866 | 28332837 | 22332284 | 13753306 | 6020022 |  |
|  | **35** | 33078795 | 32938624 | 32745368 | 32422270 | 31748412 | 30099052 | 26196038 | 19153920 |  |
|  | **40** | 33122239 | 32991368 | 32817764 | 32553732 | 32110294 | 31304331 | 29648886 | 26058143 |  |
|  | **45** | 33143961 | 33016396 | 32847712 | 32594300 | 32180995 | 31475923 | 30151858 | 27368051 |  |
|  | **50** | 33156548 | 33030651 | 32864327 | 32614758 | 32209755 | 31527447 | 30275941 | 27691854 |  |
|  | **55** | 33164354 | 33039557 | 32874563 | 32626842 | 32225251 | 31549977 | 30318070 | 27789173 |  |
|  | **60** | 33169660 | 33045587 | 32881474 | 32634814 | 32234819 | 31562363 | 30336583 | 27823570 |  |
|  | **65** | 33173689 | 33049980 | 32886518 | 32640732 | 32241752 | 31570642 | 30347225 | 27839322 |  |
|  | **70** | 33176867 | 33053480 | 32890391 | 32645099 | 32246896 | 31576773 | 30354558 | 27848926 |  |
|  | **75** | 33179325 | 33056194 | 32893389 | 32648473 | 32250739 | 31581263 | 30359986 | 27855494 |  |
|  | **80** | 33181126 | 33058233 | 32895666 | 32651015 | 32253731 | 31584848 | 30364154 | 27860570 |  |
|  | **85** | 33182580 | 33059832 | 32897574 | 32653202 | 32256188 | 31587801 | 30367726 | 27864621 |  |
|  | **90** | 33183818 | 33061186 | 32899154 | 32654972 | 32258218 | 31590172 | 30370518 | 27867775 |  |
|  | **95** | 33184918 | 33062370 | 32900536 | 32656508 | 32259928 | 31592119 | 30372739 | 27870398 |  |
