# Supplementary material for: Simultaneous testing of rule- and model-based approaches for runs of homozygosity detection opens up a window into genomic footprints of selection in pigs
Source: BMC Genomics. 2022 Aug 6;23:564. doi: 10.1186/s12864-022-08801-4 (PMC9357325; doi:10.1186/s12864-022-08801-4)
Supplement: Supplementary file 2 — Additional file 2: Table S2. Filtering for mean read depth and missing genotypes. [file 12864_2022_8801_MOESM2_ESM.docx]

Table S2. Filtering for mean read depth and missing genotypes. The number of SNPs after filtering for minimum mean read depth (min-mean-DP) and maximum number of missing genotypes (max-missing-count) is displayed.

|  |  | **max-missing-count** | | | | | |
| --- | --- | --- | --- | --- | --- | --- | --- |
|  |  | **8** | **10** | **12** | **14** | **16** | **18** |
| **min-mean-DP** | **10** | 31820357 | 32174192 | 32387193 | 32513596 | 32580304 | 32613313 |
|  | **12** | 31679791 | 31966034 | 32112832 | 32184866 | 32214370 | 32227594 |
|  | **14** | 31279931 | 31471302 | 31551775 | 31584591 | 31597187 | 31604250 |
|  | **16** | 30361555 | 30459531 | 30494400 | 30509143 | 30516591 | 30522014 |
|  | **18** | 28041849 | 28077895 | 28092479 | 28101335 | 28107240 | 28112033 |
|  | **20** | 22291891 | 22307010 | 22316387 | 22323649 | 22328978 | 22333402 |
|  | **22** | 12219273 | 12230042 | 12238027 | 12244558 | 12249447 | 12253621 |
|  | **24** | 3179054 | 3188301 | 3195500 | 3201479 | 3206012 | 3209986 |
|  | **26** | 471598 | 479911 | 486542 | 492137 | 496438 | 500265 |
|  | **28** | 192813 | 200419 | 206579 | 211869 | 216000 | 219701 |
|  | **30** | 142866 | 149975 | 155764 | 160782 | 164776 | 168376 |
