# Supplementary material for: Simultaneous testing of rule- and model-based approaches for runs of homozygosity detection opens up a window into genomic footprints of selection in pigs
Source: BMC Genomics. 2022 Aug 6;23:564. doi: 10.1186/s12864-022-08801-4 (PMC9357325; doi:10.1186/s12864-022-08801-4)
Supplement: Supplementary file 3 — Additional file 3: Table S3. Number of ROHs detected with PLINK for different scanning window parameters. [file 12864_2022_8801_MOESM3_ESM.docx]

Table S3. Number of ROHs detected with PLINK for different scanning window parameters. The number of detected ROHs, the total length of all ROHs, the minimum and maximum ROH length as well as the average length are displayed for custom and default parameter settings with varying scanning window sizes (homozyg-window-snp) and scanning window-thresholds (homozyg-window-threshold).

| **homozyg-snp [SNPs]** | **homozyg-kb [kb]** | **homozyg-density [kb/SNP]** | **homozyg-window-snp [SNPs]** | **homozyg-window-threshold** | **number of ROHs** | **total ROH lengths [kb]** | **average ROH length [kb]** | **min ROH length [kb]** | **max ROH length [kb]** | **parameter set** |
| --- | --- | --- | --- | --- | --- | --- | --- | --- | --- | --- |
| 120 | 9.93 | 0.08 | 20 | 0.25 | 297019 | 11169400,00 | 36,84 | 9,93 | 1192,31 | custom |
| 120 | 9.93 | 0.08 | 30 | 0.16 | 294243 | 10903500,00 | 36,25 | 9,93 | 1149,14 | custom |
| 120 | 9.93 | 0.08 | 40 | 0.12 | 291345 | 10663300,00 | 35,76 | 9,93 | 1149,13 | custom |
| 120 | 9.93 | 0.08 | 50 | 0.1 | 288424 | 10445600,00 | 35,36 | 9,93 | 1149,13 | custom |
| 120 | 9.93 | 0.08 | 60 | 0.08 | 285086 | 10237400,00 | 35,03 | 9,93 | 1149,13 | custom |
| 120 | 9.93 | 0.08 | 70 | 0.07 | 281692 | 10048100,00 | 34,76 | 9,93 | 1149,13 | custom |
| 120 | 9.93 | 0.08 | 80 | 0.06 | 278528 | 9869361,00 | 34,51 | 9,93 | 1149,13 | custom |
| 120 | 9.93 | 0.08 | 90 | 0.05 | 275674 | 9705947,00 | 34,27 | 9,93 | 1149,13 | custom |
| 120 | 9.93 | 0.08 | 100 | 0.05 | 272623 | 9550180,00 | 34,07 | 9,93 | 1149,13 | custom |
| 120 | 9.93 | 0.08 | 110 | 0.04 | 270077 | 9410630,00 | 33,86 | 9,93 | 1149,13 | custom |
| 120 | 9.93 | 0.08 | 120 | 0.04 | 267419 | 9271380,00 | 33,68 | 9,93 | 1149,13 | custom |
| 120 | 9.93 | 0.08 | 130 | 0.03 | 265406 | 9225270,00 | 33,80 | 9,93 | 1192,34 | custom |
| 120 | 9.93 | 0.08 | 140 | 0.03 | 262327 | 9019990,00 | 33,36 | 9,93 | 1149,13 | custom |
| 120 | 9.93 | 0.08 | 150 | 0.03 | 258547 | 8889690,00 | 33,34 | 9,93 | 1149,13 | custom |
| 100 | 1000 | 50 | 20 | 0.25 | 28 | 32527,90 | 744,28 | 1000,07 | 1694,24 | default |
| 100 | 1000 | 50 | 30 | 0.16 | 26 | 30124,10 | 632,33 | 1000,07 | 1694,24 | default |
| 100 | 1000 | 50 | 40 | 0.12 | 24 | 27413,60 | 614,62 | 1000,07 | 1694,24 | default |
| 100 | 1000 | 50 | 50 | 0.10 | 23 | 26085,20 | 611,08 | 1000,07 | 1694,24 | default |
| 100 | 1000 | 50 | 60 | 0.08 | 21 | 23917,10 | 558,24 | 1000,04 | 1694,24 | default |
| 100 | 1000 | 50 | 70 | 0.07 | 21 | 23917,00 | 558,23 | 1000,04 | 1694,24 | default |
| 100 | 1000 | 50 | 80 | 0.06 | 21 | 23916,90 | 558,23 | 1000,04 | 1694,24 | default |
| 100 | 1000 | 50 | 90 | 0.05 | 21 | 23916,90 | 558,23 | 1000,04 | 1694,24 | default |
| 100 | 1000 | 50 | 100 | 0.05 | 21 | 23911,30 | 558,09 | 1000,04 | 1694,24 | default |
| 100 | 1000 | 50 | 110 | 0.04 | 21 | 23911,10 | 558,09 | 1000,04 | 1694,24 | default |
| 100 | 1000 | 50 | 120 | 0.04 | 20 | 22848,60 | 560,61 | 1000,04 | 1694,24 | default |
| 100 | 1000 | 50 | 130 | 0.03 | 22 | 25197,80 | 560,37 | 1000,12 | 1694,33 | default |
| 100 | 1000 | 50 | 140 | 0.03 | 20 | 22712,90 | 559,26 | 1000,04 | 1566,54 | default |
| 100 | 1000 | 50 | 150 | 0.03 | 19 | 21492,40 | 498,23 | 1000,04 | 1566,54 | default |
