# Supplementary material for: Simultaneous testing of rule- and model-based approaches for runs of homozygosity detection opens up a window into genomic footprints of selection in pigs
Source: BMC Genomics. 2022 Aug 6;23:564. doi: 10.1186/s12864-022-08801-4 (PMC9357325; doi:10.1186/s12864-022-08801-4)
Supplement: Supplementary file 5 — Additional file 5: Table S5. Number of ROHs for tolerated heterozygous and missing SNPs in PLINK. [file 12864_2022_8801_MOESM5_ESM.docx]

**Table S5. Number of ROHs for tolerated heterozygous and missing SNPs in PLINK.** The number of detected ROHs, the total length of all ROHs, the minimum and maximum ROH length as well as the average length are displayed for PLINK_A and PLINK_B with a varying maximum number of heterozygous SNPs (homozyg-window-het) and missing SNPs allowed per scanning window (homozyg-window-missing).

| **homozyg-snp [SNPs]** | **homozyg-kb [kb]** | **homozyg-window-snp [SNPs]** | **homozyg-window-het** | **homozyg-window-missing** | **homozyg-window-threshold** | **number of ROHs** | **total ROH lengths [kb]** | **average ROH length [kb]** | **min ROH length [kb]** | **max ROH length [kb]** | **parameter set** |
| --- | --- | --- | --- | --- | --- | --- | --- | --- | --- | --- | --- |
| 20 | 1.6 | 20 | 0 | 3 | 0.25 | 1348464 | 11435678 | 8.76 | 1.6 | 760.59 | PLINK_A |
| 20 | 1.6 | 20 | 1 | 3 | 0.25 | 1092585 | 13257799 | 13.22 | 1.6 | 1107.18 | PLINK_A |
| 20 | 1.6 | 20 | 2 | 3 | 0.25 | 1116346 | 14189048 | 14.52 | 1.6 | 1467.72 | PLINK_A |
| 20 | 1.6 | 20 | 3 | 3 | 0.25 | 1203343 | 14988039 | 14.44 | 1.6 | 2123.08 | PLINK_A |
| 20 | 1.6 | 20 | 0 | 5 | 0.25 | 1269707 | 11874565 | 9.75 | 1.6 | 760.59 | PLINK_A |
| 20 | 1.6 | 20 | 1 | 5 | 0.25 | 967206 | 13767236 | 15.40 | 1.6 | 1192.31 | PLINK_A |
| 20 | 1.6 | 20 | 2 | 5 | 0.25 | 979651 | 14734807 | 17.20 | 1.6 | 2273.49 | PLINK_A |
| 20 | 1.6 | 20 | 3 | 5 | 0.25 | 1062839 | 15564513 | 17.13 | 1.6 | 2486.73 | PLINK_A |
| 20 | 1.6 | 20 | 0 | 7 | 0.25 | 1224398 | 12128224 | 10.41 | 1.6 | 760.59 | PLINK_A |
| 20 | 1.6 | 20 | 1 | 7 | 0.25 | 895044 | 14063846 | 17.00 | 1.6 | 1387.39 | PLINK_A |
| 20 | 1.6 | 20 | 2 | 7 | 0.25 | 901701 | 15063570 | 19.18 | 1.6 | 2785.97 | PLINK_A |
| 20 | 1.6 | 20 | 3 | 7 | 0.25 | 983095 | 15931472 | 19.12 | 1.6 | 3586.19 | PLINK_A |
| 120 | 9.93 | 120 | 0 | 3 | 0.04 | 270753 | 6651940 | 23.10 | 9.93 | 760.59 | PLINK_B |
| 120 | 9.93 | 120 | 1 | 3 | 0.04 | 272724 | 8485370 | 30.17 | 9.93 | 940.73 | PLINK_B |
| 120 | 9.93 | 120 | 2 | 3 | 0.04 | 268436 | 9407350 | 34.85 | 9.93 | 1466.76 | PLINK_B |
| 120 | 9.93 | 120 | 3 | 3 | 0.04 | 262362 | 9937660 | 38.53 | 9.93 | 2123.05 | PLINK_B |
| 120 | 9.93 | 120 | 0 | 5 | 0.04 | 277566 | 7303020 | 24.84 | 9.93 | 760.59 | PLINK_B |
| 120 | 9.93 | 120 | 1 | 5 | 0.04 | 267419 | 9271380 | 33.68 | 9.93 | 1149.13 | PLINK_B |
| 120 | 9.93 | 120 | 2 | 5 | 0.04 | 256193 | 10251400 | 39.91 | 9.93 | 2273.49 | PLINK_B |
| 120 | 9.93 | 120 | 3 | 5 | 0.04 | 246151 | 10808600 | 44.85 | 9.93 | 2273.49 | PLINK_B |
| 120 | 9.93 | 120 | 0 | 7 | 0.04 | 278149 | 7707760 | 26.15 | 9.93 | 760.59 | PLINK_B |
| 120 | 9.93 | 120 | 1 | 7 | 0.04 | 257787 | 9752210 | 36.61 | 9.93 | 1192.28 | PLINK_B |
| 120 | 9.93 | 120 | 2 | 7 | 0.04 | 240718 | 10758603 | 44.34 | 9.93 | 2477.25 | PLINK_B |
| 120 | 9.93 | 120 | 3 | 7 | 0.04 | 227637 | 11328800 | 50.49 | 9.93 | 2779.06 | PLINK_B |
