# Supplementary material for: Simultaneous testing of rule- and model-based approaches for runs of homozygosity detection opens up a window into genomic footprints of selection in pigs
Source: BMC Genomics. 2022 Aug 6;23:564. doi: 10.1186/s12864-022-08801-4 (PMC9357325; doi:10.1186/s12864-022-08801-4)
Supplement: Supplementary file 13 — Additional file 13: Table S13. DNA samples used for the evaluation of SNP filtering and ROH detection parameters. [file 12864_2022_8801_MOESM13_ESM.docx]

Table S13. DNA samples used for the evaluation of SNP filtering and ROH detection parameters. For each sample the corresponding phenotype (ear, heritage breed, disease resistance), Bioproject ID, Biosample ID, SRA ID and run accession number(s) are provided.

| **Tissue** | **Sex** | **Heritage breed (HE)** | **Disease resistance (DR; T/T; GBP5: g.127301202 G>T)** |  | | **Ear type** | | **Breed** | | **Sequencer** | | **Read length** | | **single/**  **paired end** | | **Coverage**  **(Sscrofa 11.1)** | | **Bioproject** | | **Biosample** | | **Run accession** | |
| --- | --- | --- | --- | --- | --- | --- | --- | --- | --- | --- | --- | --- | --- | --- | --- | --- | --- | --- | --- | --- | --- | --- | --- |
| hair | f | NA | NA |  | NA | | LandracexYorkshirexPietrain | | NovaSeq 6000 | | 150bp | | paired | | 35.26X | | PRJNA795885 | | SAMN24791651 | | SRR17653895 | |  |
| hair | f | NA | NA |  | NA | | Husum Red Piedx Angeln Saddleback | | NovaSeq 6000 | | 150bp | | paired | | 31.41X | | PRJNA795885 | | SAMN24791884 | | SRR17653894 | |  |
| hair | f | HE | NA |  | prick | | Kune Kune | | NovaSeq 6000 | | 150bp | | paired | | 37.98X | | PRJNA795885 | | SAMN24792119 | | SRR17653893 | |  |
| muscle | f | NA | DR |  | NA | | Angeln SaddlebackxMangalitza | | NovaSeq 6000 | | 150bp | | paired | | 29.60X | | PRJNA795885 | | SAMN24792187 | | SRR17653892 | |  |
| hair | f | NA | NA |  | lop | | Husum Red Pied | | NovaSeq 6000 | | 150bp | | paired | | 41.08X | | PRJNA795885 | | SAMN24792221 | | SRR17653901 | |  |
| hair | f | NA | NA |  | lop | | Turopolje | | NovaSeq 6000 | | 150bp | | paired | | 38.11X | | PRJNA795885 | | SAMN24792296 | | SRR17653900 | |  |
| blood | f | NA | NA |  | NA | | MinipigxMangalitza | | NovaSeq 6000 | | 150bp | | paired | | 46.32X | | PRJNA795885 | | SAMN24792411 | | SRR17653899 | |  |
| hair | m | NA | NA |  | lop | | Mangalitza | | NovaSeq 6000 | | 150bp | | paired | | 39.32X | | PRJNA795885 | | SAMN24792488 | | SRR17653898 | |  |
| ear | f | NA | DR |  | prick | | Minipig | | NovaSeq 6000 | | 150bp | | paired | | 39.53X | | PRJNA795885 | | SAMN24792530 | | SRR17653897 | |  |
| skin | f | NA | DR |  | prick | | Mini-Lewe | | NovaSeq 6000 | | 150bp | | paired | | 42.21X | | PRJNA795885 | | SAMN24792587 | | SRR17653896 | |  |
| blood | NA | NA | NA |  | lop | | Bentheim Black Pied | | Hiseq 2000 | | 100bp | | paired | | 16.58X | | PRJEB9922 | | SAMEA3497826 | | ERR977195, ERR977196 | |  |
| muscle | m | HE | NA |  | lop | | Duroc | | Hiseq 2000 | | 101bp | | paired | | 16.47X | | PRJNA260763 | | SAMN03031138 | | SRR1577872 | |  |
| blood | NA | HE | NA |  | lop | | Gloucester Old Spot | | Hiseq 2000 | | 101bp | | paired | | 18.55X | | PRJEB9922 | | SAMEA3497841 | | ERR977244, ERR977245 | |  |
| blood | f | NA | DR |  | prick | | Goettingen Minipig | | Hiseq 2000 | | 100bp | | paired | | 16.17X | | PRJEB27654 | | SAMEA4828321 | | ERR2744283 | |  |
| muscle | m | HE | NA |  | lop | | Iberian | | Hiseq 1500 | | 101bp | | paired | | 15.67X | | PRJNA320525 | | SAMN03421607 | | SRR1917381 | |  |
| liver | f | HE | DR |  | NA | | Meishan | | Hiseq 2000 | | 100bp | | paired | | 20.00X | | PRJNA309108 | | SAMN04440481 | | SRR3123403 | |  |
| blood | NA | NA | NA |  | prick | | Pietrain | | Hiseq 2000 | | 100bp | | paired | | 16.17X | | PRJEB9326 | | SAMEA3376939 | | ERR875320, ERR875321 | |  |
| blood | m | NA | DR |  | prick | | Wuzhishan minipig | | Hiseq 2000 | | 150bp | | paired | | 16.36X | | PRJNA438040 | | SAMN08689107 | | SRR6832872 | |  |
| blood | NA | HE | NA |  | prick | | Yorkshire | | Hiseq 2000 | | 101bp | | paired | | 17.34X | | PRJNA260763 | | SAMN03031189 | | SRR1581135 | |  |
| blood | f | NA | NA |  | prick | | Yucatan minipig | | Hiseq 2000 | | 101bp | | paired | | 17.70X | | PRJNA260763 | | SAMN03031169 | | SRR1581055 | |  |
